# A ubiquitin-based effector-to-inhibitor switch coordinates early brain, craniofacial, and skin development

**DOI:** 10.1038/s41467-023-40223-y

**Published:** 2023-07-26

**Authors:** Anthony J. Asmar, Shaun R. Abrams, Jenny Hsin, Jason C. Collins, Rita M. Yazejian, Youmei Wu, Jean Cho, Andrew D. Doyle, Samhitha Cinthala, Marleen Simon, Richard H. van Jaarsveld, David B. Beck, Laura Kerosuo, Achim Werner

**Affiliations:** 1 Stem Cell Biochemistry Unit, National Institute of Dental and Craniofacial Research, National Institutes of Health, Bethesda, MD 20892 USA; 2 Neural Crest Development & Disease Unit, National Institute of Dental and Craniofacial Research, National Institutes of Health, Bethesda, MD 20892 USA; 3 NIDCR Imaging Core, National Institute of Dental and Craniofacial Research, National Institutes of Health, Bethesda, MD 20892 USA; 4 Department of Genetics, University Medical Center Utrecht, Utrecht, the Netherlands; 5 Division of Rheumatology, Department of Medicine, New York University Grossman School of Medicine, New York, NY USA; 6 Center for Human Genetics and Genomics, New York University Grossman School of Medicine, New York, NY USA

**Keywords:** Protein-protein interaction networks, Neurulation, Neural tube defects, Ubiquitins, Differentiation

## Abstract

The molecular mechanisms that coordinate patterning of the embryonic ectoderm into spatially distinct lineages to form the nervous system, epidermis, and neural crest-derived craniofacial structures are unclear. Here, biochemical disease-variant profiling reveals a posttranslational pathway that drives early ectodermal differentiation in the vertebrate head. The anteriorly expressed ubiquitin ligase CRL3-KLHL4 restricts signaling of the ubiquitous cytoskeletal regulator CDC42. This regulation relies on the CDC42-activating complex GIT1-βPIX, which CRL3-KLHL4 exploits as a substrate-specific co-adaptor to recognize and monoubiquitylate PAK1. Surprisingly, we find that ubiquitylation converts the canonical CDC42 effector PAK1 into a CDC42 inhibitor. Loss of CRL3-KLHL4 or a disease-associated *KLHL4* variant reduce PAK1 ubiquitylation causing overactivation of CDC42 signaling and defective ectodermal patterning and neurulation. Thus, tissue-specific restriction of CDC42 signaling by a ubiquitin-based effector-to-inhibitor is essential for early face, brain, and skin formation, revealing how cell-fate and morphometric changes are coordinated to ensure faithful organ development.

## Abstract (web summary)

The molecular mechanisms ensuring early face, brain, and skin formation are unclear. Here, the authors uncover a posttranslational pathway that controls cytoskeletal signaling circuits to coordinate ectodermal patterning and neurulation.

## Introduction

During early development, the ectoderm layer of vertebrate embryos undergoes neurulation, involving drastic morphological changes to form the neural tube, while it is simultaneously patterned into three distinct domains. The lateral non-neural ectoderm domain will form the protective epidermis, and the medial neural plate domain gives rise to the central nervous system (CNS) that forms the brain in the head and the spinal cord in the posterior body. In between these two domains is the neural plate border, which forms the cranial placodes and the neural crest cells that will generate multiple derivatives, including the peripheral nervous system and, in the head region, the craniofacial skeleton^1^. A failure to faithfully establish and differentiate ectodermal domains results in a range of congenital birth defects affecting the development of the central and peripheral nervous system, craniofacial structures, and the skin^2–5^. Extensive studies in different model systems have identified key morphogens gradients in the embryo that induce major transcriptional changes driving ectodermal patterning into the respective domains^6–17^. Yet, the molecular mechanisms that determine these initial cell-fate decisions and coordinate them with morphometric cell changes to spatially separate domains within the neurulating ectoderm have remained elusive^18^.

**Fig. 1:**
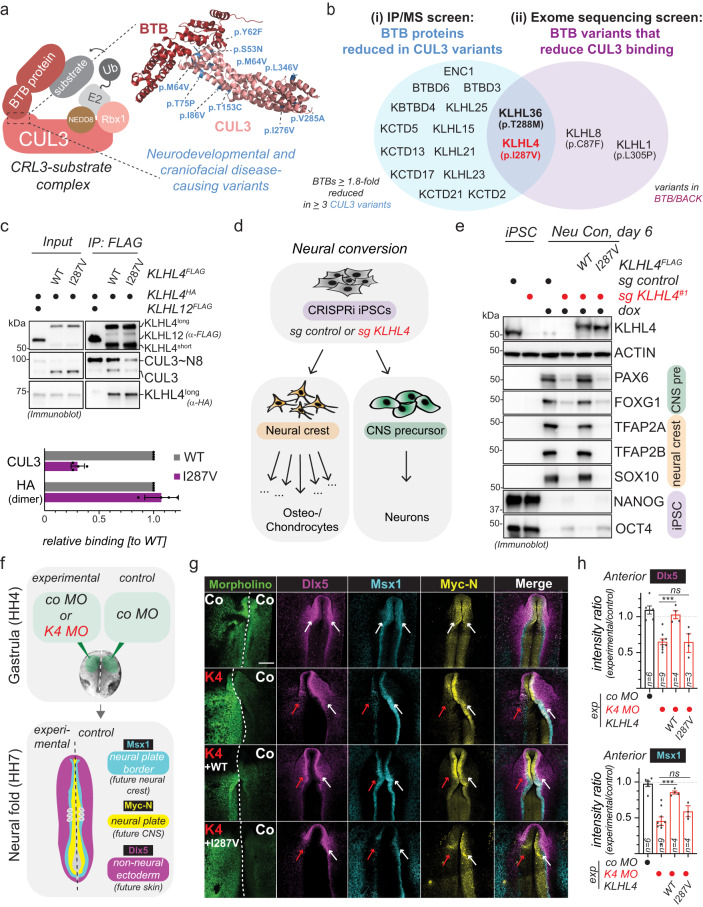
CRL3-KLHL4 controls ectodermal patterning and neurulation in the vertebrate head. **a** Model of a substrate-engaged CRL3 complex with an example crystal structure of a CUL3-BTB interface (pdb: 4APF) highlighting the positions of developmental disease-causing missense variants in CUL3. **b** Schematic overview of the IP/MS (i) and the exome sequencing screen (ii) for novel CRL3-BTB complexes with functions during ectodermal differentiation, identifying CRL3-KLHL4 and CRL3-KLHL36 as hits. **c** Immunoblot analysis of FLAG-IP fractions from HEK293T cells reveals that the KLHL4 p.I287V patient variant reduces CUL3 binding. Ectopically expressed KLHL4 is present in two isoforms (short and long), likely originating from differential usage of start codons (Supplementary Fig. 2n). *n* = 4 biological replicates, error bars = s.d., CUL3: ****P < 0.0001, HA: *P* = 0.3640, unpaired *t* test. **d** Schematic overview of the neural conversion differentiation paradigm. CNS = central nervous system. **e** CRL3-KLHL4 is required for neural conversion. Control or KLHL4-depleted CRISPRi iPSCs expressing sgRNA-resistant and doxycycline-inducible wild-type (WT) or patient variant (I287V) KLHL4^FLAG^ were treated with doxycycline (dox) and neural conversion for 6 days as indicated. Immunoblotting shows decreased expression of CNS and neural crest markers upon KLHL4 depletion that is rescued by WT but not p.I287V KLHL4. ACTIN = loading control. Immunoblots = 2 biological replicates. **f** Fluorescein-labeled translation-blocking morpholinos were electroporated into the ectoderm at gastrula stage (HH4) by treating one side with a KLHL4 Morpholino (K4) and the other side with a control morpholino (Co). The embryos were incubated until the neural folds stage (HH7-8). The experimental side was compared to the control side, and control embryos were treated with Co on both sides. **g** Loss of KLHL4 reduces anterior expression *Dlx5*, *Msx1*, and *Myc-N* as shown by HCR fluorescent in situ hybridization. Loss of KLHL4 is rescued by co-expression of WT KLHL4, but not with the patient mutation KLHL4 I287V. The arrows point to the neural folds on the experimental (red) and control (white) side. **h** Quantification of fluorescence intensities (*Dlx5* and *Msx1*: *n* = 4–9 embryos as indicated by the number in the respective bar, *Dlx5*: *P* = 0.000237; *Msx1*: *P* = 0.0004, one-way ANOVA). Scale bar = 250 μm.

Cullin-RING E3 ubiquitin ligases (CRLs) are a large class of ~300 modular enzymes that control critical aspects of human development and physiology^19–22^. The CRL3 sub-family of these enzymes consists of a stable catalytic core complex, CUL3-RBX1, which is paired with one of ~120 interchangeable substrate adaptors that contain a BTB domain^23–25^. While the BTB domain connects the adaptor to CUL3-RBX1, other dedicated domains in the adaptor (e.g., KELCH repeats) recognize specific substrates (Fig. 1a). The assembly and disassembly of particular CRL3-substrate complexes is tightly regulated within cells and involves reversible modification with NEDD8 (neddylation), the substrate adaptor exchange factor CAND1^26–32^, and co-adaptors to help recruit substrates and to coordinate the assembly process^33–35^. CRL3s often catalyze non-degradative monoubiquitylation^22,34–37^ to control organismal development and tissue homeostasis^38–40^. Specifically, CRL3s play crucial roles in ectodermal differentiation, as evidenced by the fact that more than 20 different loss-of-function variants in the *CUL3* locus are associated with neurodevelopmental and craniofacial diseases^41^ (Supplementary Data 1). However, how particular CRL3 complexes control the early stages of ectodermal patterning and neural tube formation remains unknown.

Here, by combining systematic human developmental disease-variant screening with biochemical, iPSC, and chick embryo approaches, we identify CUL3 in complex with its substrate adaptor KLHL4 (CRL3-KLHL4) as central to a novel posttranslational pathway that is essential for neurulation and patterning of the ectodermal domains. We find that monoubiquitylation by CRL3-KLHL4 catalyzes an effector-to-inhibitor conversion to restrict cytoskeletal signaling of the small GTPase CDC42 in the developing vertebrate head. Our data thus uncover a previously unrecognized principle of small GTPase signaling that coordinates cell-fate and morphometric changes to establish the future skin, brain, and craniofacial skeleton and, upon dysregulation, contributes to craniofacial and brain malformations.

## Results

### CRL3-KLHL4 controls ectodermal patterning and neurulation

To identify CRL3s with previously unrecognized functions during ectodermal differentiation, we biochemically profiled known or candidate neurodevelopmental and craniofacial disease-causing variants in the CUL3-BTB interface (Fig. 1a). We identified KLHL4 and KLHL36 as the only BTB adaptors that were both (i) reduced in immunoprecipitation (IP) fractions of several disease-associated CUL3 variants and (ii) for which we found variants in patients with undiagnosed developmental diseases that reduced their interaction with CUL3 (Fig. 1b, Supplementary Fig. 1a, b, Supplementary Fig. 2a–m, Supplementary Data 2*,* and Supplementary Data 3). For a more detailed description of the human disease-variant screen refer to Supplementary Note 1. Given its previous connection to craniofacial development^42,43^, we focused on *KLHL4* for follow-up studies. The maternally inherited hemizygous missense variant in KLHL4 (NM_019117.4: c.859 A > G [p.(Ile287Val)] that we identified in our exome sequencing screen is present in a male proband, exhibiting severe intellectual disability and multiple congenital anomalies, including brain malformations (for a more detailed patient description refer to Supplementary Note 2). The KLHL4 p.I287V variant significantly reduced binding to unmodified and neddylated CUL3, while not affecting KLHL4 dimerization (Fig. 1c and Supplementary Fig. 2k).

To test for a function of CRL3-KLHL4 during ectodermal differentiation, we generated control and KLHL4-depleted CRIPSRi iPSCs^44^ and subjected these cell lines to neural conversion, an in vitro protocol that induces neuroepithelial differentiation into cells of the central nervous system and the neural crest^45^ (Fig. 1d). Utilizing self-made monoclonal antibodies against KLHL4, we observed a marked reduction of KLHL4 protein levels during neural conversion of control iPSCs (Fig. 1e), raising the possibility that KLHL4 could play a role during early stages of ectodermal differentiation. Indeed, while we observed no substantial differences in cell growth or the expression of pluripotency markers OCT4 and NANOG in the ground state, there was a notable defect in forming  neural and neural crest derivatives when comparing control and KLHL4-depleted iPSCs (Fig. 1e and Supplementary Fig. 2o). This was apparent by the loss of neural crest markers (including SOX10 and TFAP2A/B), reduced expression of CNS markers (including the forebrain marker FOXG1 and the neural stem cell marker PAX6), and failure to downregulate OCT4 and NANOG, as evidenced by immunoblotting, quantitative polymerase chain reaction (qPCR), and immunofluorescence (Fig. 1e and Supplementary Fig. 3a, b). We could further corroborate these findings during an alternative in vitro protocol, known as embryoid body formation. While depletion of KLHL4 using two different siRNAs had no obvious impact on stem cell maintenance or embryoid body formation of human embryonic stem cells (Supplementary Fig. 4a–d), KLHL4-depleted embryoid bodies were deficient in cell outgrowth and differentiation into neural progenitor cells and neurons when cultured in a matrigel-based 3D matrix, as evidenced by light microscopy, qPCR, and immunofluorescence analysis (Supplementary Fig. 4c–f). Importantly, KLHL4-depletion-induced phenotypes during neural conversion could be rescued by wild-type (WT) KLHL4, but not by the CUL3-binding-deficient KLHL4 p.I287V patient variant (Fig. 1e and Supplementary Fig. S3a, b), suggesting that the ubiquitylation activity of CRL3-KLHL4 is required to support ectodermal differentiation of pluripotent stem cells into the CNS and the neural crest.

To corroborate the above findings in vivo, we next performed experiments in chick embryos, an amniote model with a high resemblance to human development^46^. In situ hybridization analysis revealed *Klhl4* to be exclusively expressed in the ectoderm and restricted to the anterior part of the developing embryo that will form the head (Supplementary Fig. 5a), suggesting a tissue-specific role of CRL3-KLHL4 during ectodermal cell-fate specification. To test the role of KLHL4 in the early ectoderm, we used a translation-blocking morpholinos against *KLHL4* (K4 MO) that was injected and electroporated on one side of the gastrula stage embryos while the contralateral side was injected with a control morpholino (co MO), and the results were compared to embryos that were treated with the co MO on both sides (Fig. 1f). We found that loss of KLHL4 severely interfered with ectodermal lineage commitment of all respective domains (Fig. 1g and Supplementary Fig. 5b), as the expression of the neural plate/CNS marker *MycN*, the neural plate border marker *Msx1*, and the non-neural ectoderm and early epidermal marker *Dlx5*, were significantly reduced in the KLHL4 MO-injected side as compared to the side with the control MO as shown by multichannel fluorescent in situ hybridization from whole embryos (Fig. 1g, h and Supplementary Fig. 5b). Consistent with the expression pattern of KLHL4, these phenotypes were only present in the anterior, but not in the posterior part of the embryo (Supplementary Fig. 5b–d). Furthermore, analysis of corresponding anterior transverse sections revealed that depletion of KLHL4 prevented proper neurulation. The folding of the neural plate was incomplete on the KLHL4 knockdown side and lacked the dorsolateral hinge point^47^, resulting in a significantly longer distance from the apex of the neural fold to the notochord than compared to the control side (Supplementary Fig. 5e, f). The sections also revealed that loss of KLHL4 significantly compromised the establishment of defined borders between the distinct ectodermal domains, measured as an increase in the overlap of the expression markers representing the distinct domains (Supplementary Fig. 5g). Consistently, we detected a significant increase in the length of the neural plate border domain upon KLHL4 depletion when measured by immunostaining for another neural plate border marker, Pax7, which also showed a significantly lower protein expression level on the K4 MO side (Supplementary Fig. 5h–j). Next, we analyzed the embryos at a later developmental stage at the end of neurulation to investigate whether changes at the neural plate border resulted in defects in neural crest cell specification. Indeed, consistent with our findings from our in vitro differentiation experiments (Fig. 1e and Supplementary Fig. 3a), in situ hybridization revealed that loss of KLHL4 caused a reduction in the expression of *Pax7* as well as the mature neural crest markers *FoxD3* and *Sox10* at the onset of their migration from the dorsal neural tube (Supplementary Fig. 5k). Importantly, the KLHL4 MO knockdown phenotype was rescued with WT KLHL4, but not with the CUL3-binding-deficient KLHL4 p.I287V patient variant, which continued to show an aberrant ectodermal patterning phenotype (Fig. 1g, h). In this context it is important to note that these experiments are not designed to precisely mimic the degree of loss of KLHL4 in the patient, and the morpholino-mediated knockdown may thus result in lower KLHL4 activity and more severe differentiation phenotypes than compared to those in the patient. Taken together, these results suggest that during the early stages of embryo development, tissue-specific ubiquitylation by CRL3-KLHL4 ensures neural plate folding and cell-fate specification of the head ectoderm into functionally distinct domains, and impairment of this activity through mutations likely contributes to neurodevelopmental and craniofacial diseases.

### Identification of the GIT-PIX-PAK signaling module as interactors and candidate substrates of CRL3-KLHL4

**Fig. 2:**
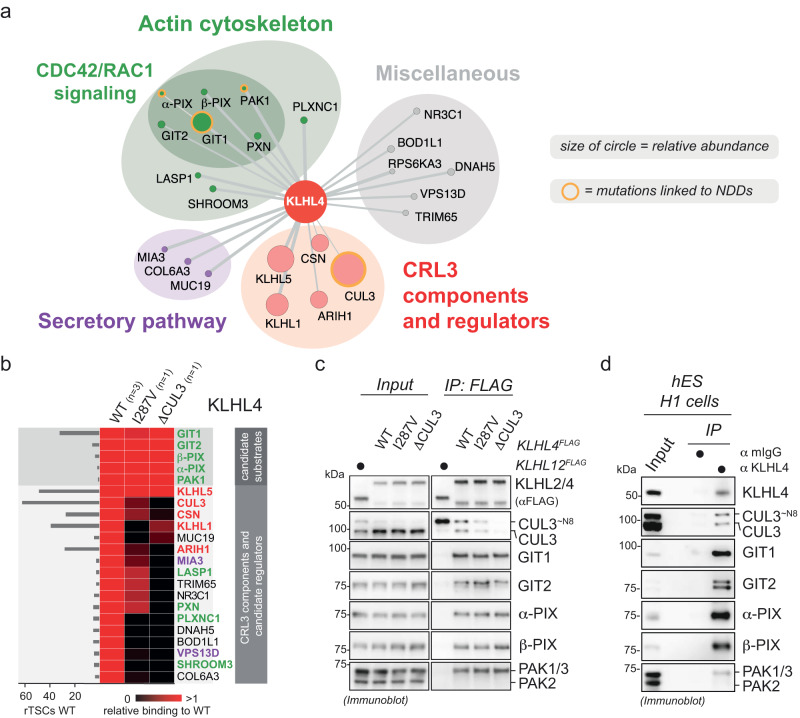
Identification of components of the GIT-PIX-PAK signaling module as interactors and candidate substrates of CRL3-KLHL4. **a** KLHL4 interacts with many actin cytoskeleton regulators, including components of the GIT-PIX-PAK module, which controls signaling by the small GTPases CDC42 and RAC1. High-confidence interaction partners of KLHL4 were determined by compPASS-based mass spectrometry (*n* = 3 biological replicates) and depicted in an interaction network according to their molecular function, relative abundance (size of circles), and specificity scores (thickness of lines). Proteins previously shown to cause neurodevelopmental disease (NDD) when mutated are circled in orange. **b** Components of the GIT-PIX-PAK signaling module are candidate substrates of CRL3-KLHL4. High-confidence interaction partners of wild-type KLHL4 (WT) were determined by compPASS-based mass spectrometry (*n* = 3 biological replicates) and relative total spectral counts for each high-confidence interaction partner found in WT KLHL4 IPs were compared to the ones found in one replicate of KLHL4 I287V and KLHL4ΔCUL3 IPs in a heatmap (black = no interaction, red = equal or more interaction). A quantification of normalized total spectral counts (TSCs) of three biological replicates is shown for KLHL4 WT. Color coding of protein names is from categories in panel (**a**). **c** Components of the GIT-PIX-PAK signaling module interact with CRL3-KLHL4 through the substrate adaptor, as shown by immunoblot analysis of indicated KLHL4^FLAG^ construct IPs from HEK293T cells. Immunoblots are representative of three biological replicates. **d** Endogenous KLHL4 interacts with the GIT-PIX-PAK signaling module in hESCs (H1 line), as evidence by immunoblot analysis of endogenous anti-KLHL4 IPs. mIgGs were used as IP control. Immunoblots are representative of two biological replicates.

To elucidate the molecular mechanisms by which CRL3-KLHL4 allows proper distinction of ectodermal domains and neurulation, we employed an approach that had previously allowed us to define stem cell-related signaling pathways^35,48^. We used compPASS mass spectrometry^49,50^ to identify candidate substrates of CRL3-KLHL4 (as explained in more detail in Supplementary Note 3). We determined proteins that bind to WT KLHL4, KLHL4 p.I287V patient variant, and a more severe CUL3-binding mutant (KLHL4ΔCUL3) that we generated by mutating a tyrosine in the BTB domain known to be critical for CUL3 interaction^35^. These interaction networks revealed GIT1, GIT2, α-PIX, β-PIX, and PAK1 as interactors that bound equally to WT KLHL4, KLHL4 p.I287V, and KLHL4 ΔCUL3, indicating interaction with the substrate-binding domain of KLHL4 (Fig. 2a, b and Supplementary Data 4). These proteins are components of GIT-PIX-PAK assemblies, which regulate cytoskeletal signaling by modulating the activity of the small GTPases CDC42 and RAC1^51^. We confirmed the CRL3-KLHL4 interaction with the GIT-PIX-PAK signaling module by IP/immunoblotting (Fig. 2c) and showed that the same associations occur at the endogenous level in hESCs (Fig. 2d).

### CRL3-KLHL4 uses GIT-PIX complexes as co-adaptors to recruit and multi-monoubiquitylate PAK1

**Fig. 3:**
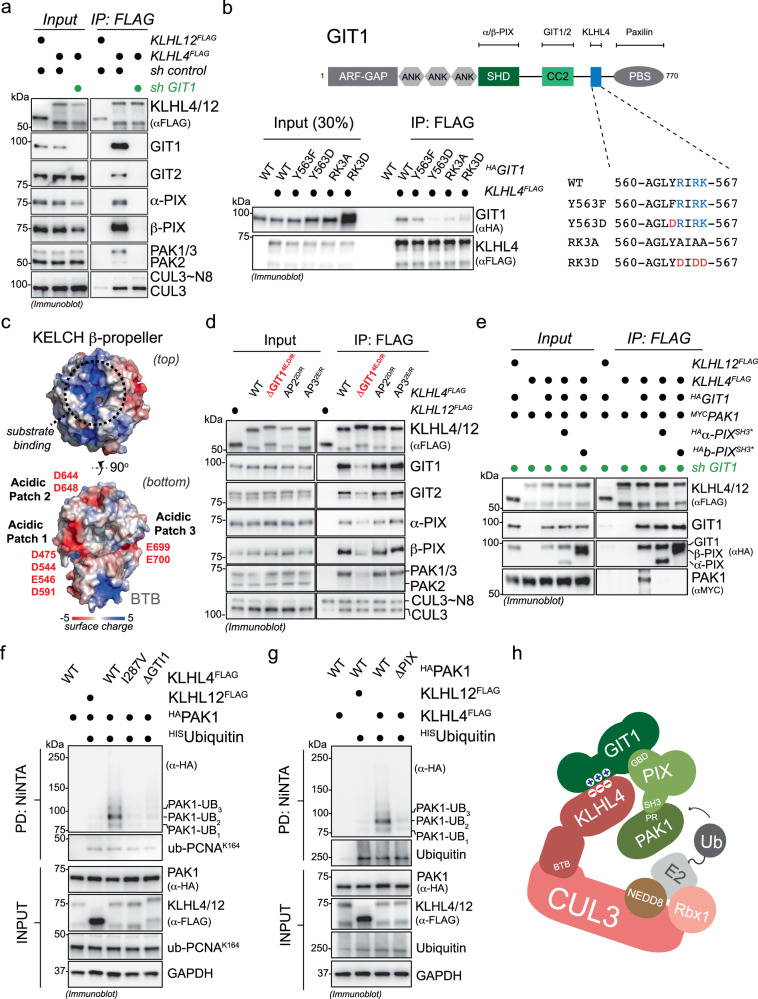
CRL3-KLHL4 interacts with the GIT-PIX-PAK signaling module through GIT1 and utilizes GIT-PIX complexes as co-adaptors to multi-monoubiquitylate PAK1. **a** CRL3-KLHL4 engages the GIT-PIX-PAK signaling module through GIT1, as evidenced by immunoblot analysis of anti-FLAG IPs from control or GIT1-depleted HEK293T cells. *n* = 2 biological replicates. **b** Three positively charged residues in GIT1 are essential for KLHL4 binding. Immunoblot analysis of anti-FLAG IPs from HEK293T cells transfected with indicated constructs. *n* = 2 biological replicates. GIT1 = protein isoform 3 on UniProt. **c** KLHL4 possesses three acidic patches on the bottom of its KLECH propeller. Depicted is a surface charge map of a structural model of KLHL4. The upper panel shows the top of the KELCH β-propeller, the canonical binding site for substrates in other BTB-KELCH proteins (indicated with black dotted circle), which exhibits a positively charged groove. The lower panel depicts the bottom of the KELCH β-propeller that contains three acidic patches. **d** Acidic patch 1 (ΔGIT1), but not acidic patch 2 or 3 (AP2 and AP3), in KLHL4 mediates binding to the GIT-PIX-PAK signaling module. Immunoblot analysis of anti-FLAG IPs from HEK293T cells transfected with indicated constructs. *n* = 3 biological replicates. **e** GIT-PIX complexes recruit PAK1 to KLHL4. GIT1-depleted HEK293T cells were co-transfected with indicated constructs, lysed, and subjected to anti-FLAG-IP followed by immunoblotting with denoted antibodies. KLHL4 can only bind PAK1 when shRNA-resistant GIT1 is expressed, and this association can be blocked by co-expression of PAK-binding-deficient PIX proteins (α/β-PIX ^SH3*^). *n* = 1 biological replicate. **f** CRL3-KLHL4 ubiquitylates PAK1 in cells. Immunoblot analysis of denaturing ^HIS^ubiquitin pulldowns from RPE-1 cells expressing denoted combinations of constructs using the indicated antibodies. PCNA = control for general ubiquitylation efficiency. *n* = 3 biological replicates. **g** PAK1 requires interactions with GIT-PIX complexes to be ubiquitylated by CRL3-KLHL4 in cells. Immunoblot analysis of denaturing ^HIS^ubiquitin pulldowns from RPE-1 cells expressing denoted combinations of constructs. ΔPIX = GIT-PIX-complex-binding-deficient PAK1. Ubiquitin = control for general ubiquitylation efficiency. *n* = 2 biological replicates. **h** Model how CRL3-KLHL4 uses GIT1-PIX complexes as co-adaptors to bind and ubiquitylate PAK1.

To determine how CRL3-KLHL4 engages the GIT-PIX-PAK signaling module, we performed a series of biochemical and cell-based experiments. IP of KLHL4 from GIT1-depleted cells showed that GIT1 was required for KLHL4 to recognize GIT2, α-PIX, β-PIX, PAK1, or PAK3, but not CUL3 (Fig. 3a and Supplementary Fig. 6a). Truncation and mutational analyses revealed three positively charged amino acids (R564, R566, and K567) in GIT1 that, when substituted with negatively charged amino acids (GIT1^RK3D^), impaired interaction with KLHL4 (Fig. 3b and Supplementary Fig. 6b, c). Only R564 is present in GIT2, the otherwise highly conserved homolog of GIT1 (Supplementary Fig. 6d), providing a molecular rationale for why we find only GIT1, and not GIT2, required for targeting the GIT-PIX-PAK module to CRL3-KLHL4 (Supplementary Fig. 6e). Through structural modeling^52^, we identified a complementary negatively charged surface on the bottom of the KLHL4 β-propeller that when disrupted by charge swap mutations (D475R, D544R, E546R, D591R) reduced binding to the GIT-PIX-PAK module (KLHL4ΔGIT1, Fig. 3c, d and Supplementary Fig. 6a), indicating that GIT1 binds KLHL4 on the opposite side of where substrate binding usually occurs in BTB-KELCH proteins^53–55^. Indeed, using recombinantly purified proteins, we were able to in vitro reconstitute binding of KLHL4 to GIT1, but not to GIT1^RK3D^ (Supplementary Fig. 6f). Collectively, our results show that CRL3-KLHL4 engages the GIT-PIX-PAK signaling module through a direct electrostatic interaction with GIT1.

Since our results revealed that the GIT1-KLHL4 binding interface is distal from the canonical substrate-binding site (Fig. 3c, d), we reasoned that this interaction might serve to recruit PIX and/or PAK proteins to the CRL3-KLHL4 complex for ubiquitin ligation. Indeed, building on previously mapped interactions of GIT-PIX-PAK module^51,56^ (Supplementary Fig. 7a), we found that ectopically expressed GIT1 increased the association of endogenous α-PIX and β-PIX to affinity-purified KLHL4^FLAG^, while KLHL4 binding-deficient GIT1^Y563D^ (Fig. 3b) sequestered these proteins and prevented their association with KLHL4 (Supplementary Fig. 7b). In addition, IP experiments from GIT1-depleted cells revealed that binding of PAK1 to KLHL4 was only supported upon rescue by expressing shRNA-resistant GIT1, and this association could be blocked by co-expression of PIX proteins (α-PIX^SH3*^, β-PIX ^SH3*^^57^) that are unable to bind PAK1 (Fig. 3e). Thus, engagement of CRL3-KLHL4 to the GIT-PIX-PAK signaling module occurs through GIT1 that binds to PIX proteins, which can further recruit PAK1 (Supplementary Fig. 7c).

To test whether the above interactions result in ubiquitylation, we expressed His-tagged ubiquitin with different components of the GIT-PIX-PAK signaling module in the absence and presence of different KLHL4 variants in cells and purified ubiquitin conjugates under denaturing conditions. Intriguingly, we found that KLHL4, but not the CUL3-binding-deficient KLHL4 p.I287V patient variant, induced robust ubiquitylation of PAK1 (Fig. 3f), while under similar experimental conditions GIT1-β-PIX complexes were not ubiquitylated in a KLHL4-dependent manner (Supplementary Fig. 8a). PAK1 ubiquitylation was not supported by KLHL4ΔGIT1, indicating that CRL3-KLHL4 requires GIT1-PIX complexes to form efficient ubiquitin ligation assemblies with PAK1 (Fig. 3f). Corroborating this notion, a PAK1 mutant unable to interact with PIX proteins (PAK1^ΔPIX^) was also deficient in KLHL4-dependent ubiquitylation (Fig. 3g and Supplementary Fig. 8b). KLHL4-dependent PAK1 modifications are likely multi-monoubiquitylation events, as we predominantly observed attachment of up to three ubiquitin molecules to lysine residues in the C-terminus of PAK1 (Fig. 3f, g and Supplementary Fig. 8c–g) and a lysine-less version of ubiquitin still supported the same pattern of modification for PAK1 (Supplementary Fig. 8h). Consistent with such multi-monoubiquitylation, which typically does not mediate proteasomal degradation, we did not detect changes in the steady state levels of PAK1 or other components of the GIT-PIX-PAK module upon loss of KLHL4 in cells (Supplementary Fig. 8i). From these results, we infer that CRL3-KLHL4 utilizes GIT-PIX complexes as co-adaptors to recruit and multi-monoubiquitylate PAK1 (Fig. 3h).

### CRL3-KLHL4 regulates ectodermal differentiation through monoubiquitylation of PAK1

**Fig. 4:**
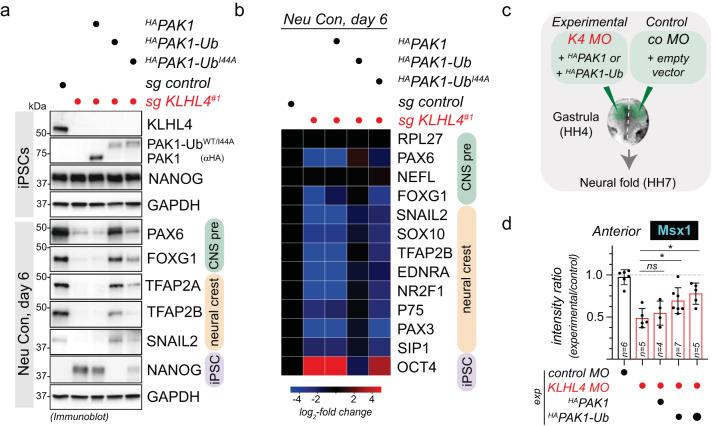
Monoubiquitylation of PAK1 controls ectodermal differentiation. **a** Ubiquitylated PAK1 can rescue neural conversion of KLHL4-deficient iPSCs. Control or KLHL4-depleted CRISPRi iPSCs were reconstituted with doxycycline-inducible wild-type ^HA^PAK1, ^HA^PAK1-Ub, or ^HA^PAK1-Ub^I44A^, treated with doxycycline (dox) and subjected to neural conversion for 6 days. Differentiation was monitored by immunoblotting using antibodies against indicated lineage markers. Immunoblots are representative of two biological replicates. **b** Ubiquitylated PAK1 can rescue neural conversion of KLHL4-deficient iPSCs, as monitored by qPCR analysis of iPSCs treated and differentiated as in panel (**a**). CNS precursor markers = green, neural crest markers = orange, and iPSC markers = purple. Marker expression was normalized to control iPSCs and RPL27 was used as endogenous control. Heatmap depicts the average of two biological replicates with three technical replicates each. **c** Experimental design for in vivo electroporations testing whether constitutively ubiquitylated PAK1 can rescue KLHL4 depletion. The results are compared to embryos with Co MO-injected on both sides prior to electroporation. **d** Morpholino-induced loss of KLHL4 phenotype is partially rescued in by co-expression of ubiquitylated PAK1, but not by the non-ubiquitylated WT PAK1, as quantified by ratio of Msx1 fluorescence intensity between experimental and control side from embryos stained with whole-mount HCR FISH, which show a significant difference between KLHL4 MO and PAK1-Ub rescue. *n* = 4–7 embryos as indicated by the number in the bar, error bars = s.e.m., PAK1-Ub low: *P* = 0.0299, PAK1-UB high: *P* = 0.0165, one-way ANOVA.

To verify that PAK1 ubiquitylation is important for the function of CRL3-KLHL4 in ectodermal development, we performed a series of iPSC differentiation experiments. First, similar to loss of KLHL4, iPSCs expressing only GIT-PIX-PAK-binding-deficient KLHL4ΔGIT1 failed to support CNS precursor and neural crest cell formation (Supplementary Fig. 9a–c). Second, the same aberrant neural conversion program was observed if we depleted GIT1, β-PIX, and PAK1/2/3, while depletion of KLHL5, GIT2, or α-PIX caused less dramatic changes (Supplementary Fig. 9d, e). These results are consistent with our findings that GIT1, but not GIT2, is required for PAK1 recruitment to KLHL4 (Supplementary Fig. 6e) and further suggest that during differentiation, it is mainly GIT1-β-PIX complexes that bridge interactions of CRL3-KLHL4 with PAK1 for ubiquitin ligation. Third, we asked whether constitutive ubiquitylation of PAK1 would rescue the KLHL4-depletion-induced phenotypes during ectodermal differentiation. To mimic substrate ubiquitylation, we utilized an approach previously described by others^33,58,59^ and fused ubiquitin to the C-terminus of PAK1 (^HA^PAK1-Ub), placing it in proximity to where we mapped the ubiquitylation sites (Supplementary Fig. 8c–g). We found that PAK1-Ub was able to restore the neural conversion program of KLHL4-depleted iPSCs, while this was not the case for its unmodified counterpart ^HA^PAK1 (Fig. 4a, b). In addition, PAK1 fused to ubiquitin mutated in its hydrophobic patch (^HA^PAK1-Ub^I44A^) was markedly reduced in its ability to rescue differentiation defects caused by loss of KLHL4. As this hydrophobic patch mutant abrogates the majority of interactions with ubiquitin-binding domains^60,61^, our results suggest that monoubiquitylated PAK1 mediates cell-fate determination at least in part through recognition by a ubiquitin-binding effector protein. Strikingly, constitutively ubiquitylated PAK1-Ub also partially rescued KLHL4-depletion-induced defects in ectodermal patterning during chick development, as quantified by the expression of the neural plate border marker *Msx1* (Fig. 4c, d and Supplementary Fig. 10a). In contrast, non-ubiquitylated PAK1 was not able to restore normal ectodermal cell-fate specification in KLHL4 MO-injected embryos and even resulted in excessive and ectopic expression of *Msx1* in the lateral ectoderm and extra-embryonic tissue in some cases (Supplementary Fig. 10a). Collectively, these results suggest that GIT1-β-PIX-dependent monoubiquitylation of PAK1 is a central mechanism underlying the function of CRL3-KLHL4 in controlling ectodermal patterning (Supplementary Fig. 10b). Consistent with our findings, genetic lesions in the GIT-PIX-PAK signaling axis are known to cause congenital diseases affecting brain and craniofacial development with phenotypic overlap to *CUL3* and *KLHL4* patients^62–65^.

### Monoubiquitylation converts PAK1 into an inhibitor of CDC42 signaling

**Fig. 5:**
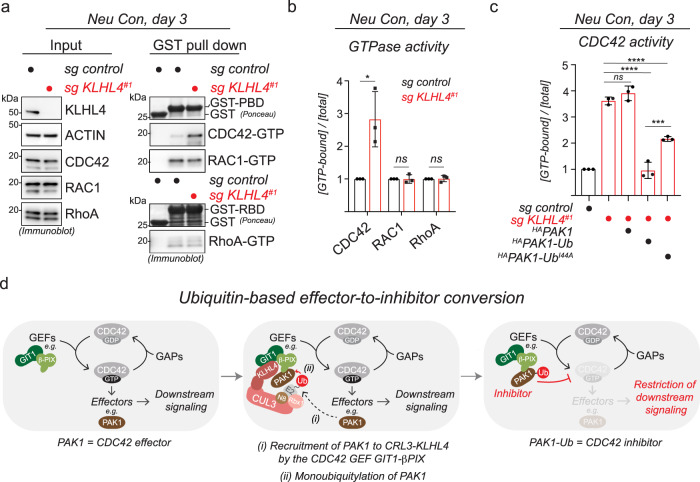
Monoubiquitylation of PAK1 restricts CDC42 signaling. **a** KLHL4 depletion increases CDC42 activity in cells undergoing early stages of neural conversion. Control or KLHL4-depleted CRISPRi iPSCs were subjected to neural conversion for 3d, lysed, and endogenous active (GTP-bound) small GTPases were affinity-purified using GST-PBD (CDC42/RAC1) or GST-RBD (RhoA) followed by immunoblotting with indicated antibodies. GST was used as a specificity control for binding. **b** Quantification of the experiment depicted in panel (**a**). Relative small GTPase activity was calculated by dividing the GTP-bound state to the total levels of each small GTPase followed by normalization to control cells. mean of *n* = 3 biological replicates, error bars = s.d., CDC42: *P* = 0.0202, RAC1: *P* = 0.9888, RhoA: *P* = 0.864, unpaired *t* test. **c** Ubiquitylated PAK1 can restore normal levels of CDC42 activity in KLHL4-deficient cells undergoing early stages of neural conversion. Control or KLHL4-depleted CRISPRi iPSCs were reconstituted with dox-inducible ^HA^PAK1, ^HA^PAK1-Ub, or ^HA^PAK1-Ub^I44A^, treated with dox and subjected to neural conversion for 3d. GTP-bound CDC42 was affinity-purified and relative CDC42 activity was calculated as described above. mean of *n* = 3 biological replicates, error bars denote s.d., PAK1: *P* = 0.4227, PAK1-Ub: *P* < 0.0001, PAK1-Ub^I44A^: *P* < 0.0001, PAK1-Ub vs. PAK1-Ub^I44A^: *P* = 0.0002, one-way ANOVA. **d** Model of how CRL3-KLHL4 restricts CDC42 signaling during ectodermal differentiation. PAK1 canonically acts as a CDC42 effector kinase but can be converted into a CDC42 inhibitor. This requires (i) recruitment of PAK1 to CRL3-KLHL4 by the CDC42 GEF GIT1-β-PIX and (ii) monoubiquitylation.

We next aimed to determine how monoubiquitylation of PAK1 regulates cellular signaling to ensure proper ectodermal patterning. The GIT-PIX-PAK axis participates in remodeling of the actin cytoskeleton through Rho-family small GTPases^51,66^. Consistent with these studies, we found that CRL3-KLHL4 regulates cell shape and actin dynamics in iPSCs and RPE cells (Supplementary Fig. 11), as indicated by KLHL4-depletion-induced changes in actin protrusions (Supplementary Fig. 11a, b) and epithelial sheet morphology with less cell packing (Supplementary Fig. 11c) and significantly increased cell spread area (Supplementary Fig. 11d–g) and also resulted in significantly slower actin flow and migration rates during wound-healing assays (Supplementary Fig. 11h–k and Supplementary Movie 1). We therefore hypothesized that CRL3-KLHL4-dependent monoubiquitylation of PAK1 might regulate RhoA, RAC1, or CDC42 signaling to ensure ectodermal cell-fate commitment. Intriguingly, using affinity pulldown of GTP-bound GTPases from cells, we found that KLHL4 depletion significantly increased CDC42 activity in iPSC undergoing early stages of neural conversion, while RhoA or RAC1 were not affected (Fig. 5a, b). This effect could be rescued by ectopic expression of WT KLHL4, but not by the CUL3-binding- or GIT-PIX-PAK-binding-deficient variants of KLHL4 (I287V and ΔGIT1, respectively) (Supplementary Fig. 12a, b), suggesting that CRL3-KLHL4-dependent ubiquitylation of PAK1 is a means to restrict CDC42 activity. Indeed, normal levels of CDC42 activation in KLHL4-depleted cells could be restored by expression of constitutively ubiquitylated PAK1 (^HA^PAK1-Ub), but not its unmodified counterpart (^HA^PAK1) and only partially by PAK1 fused to the ubiquitin hydrophobic patch mutant (^HA^PAK1-Ub^I44A^) (Fig. 5c and Supplementary Fig. 12c). Similarly, co-depletion of the GTPase exchange factors (GEFs) α-PIX and β-PIX in KLHL4-deficient cells, re-established CDC42-GTP levels to that of control cells (Supplementary Fig. 12d, e). From these experiments, we conclude that during ectodermal differentiation, ubiquitylated PAK1, likely through interaction with a ubiquitin-binding effector protein, restricts CDC42 activity by counteracting α/β-PIX-mediated GDP-to-GTP exchange (Fig. 5d), revealing that monoubiquitylation can switch a canonical CDC42 effector^67,68^ into an inhibitor.

### Monoubiquitylation of PAK1 balances anterior CDC42 signaling to coordinate ectodermal patterning and neurulation

**Fig. 6:**
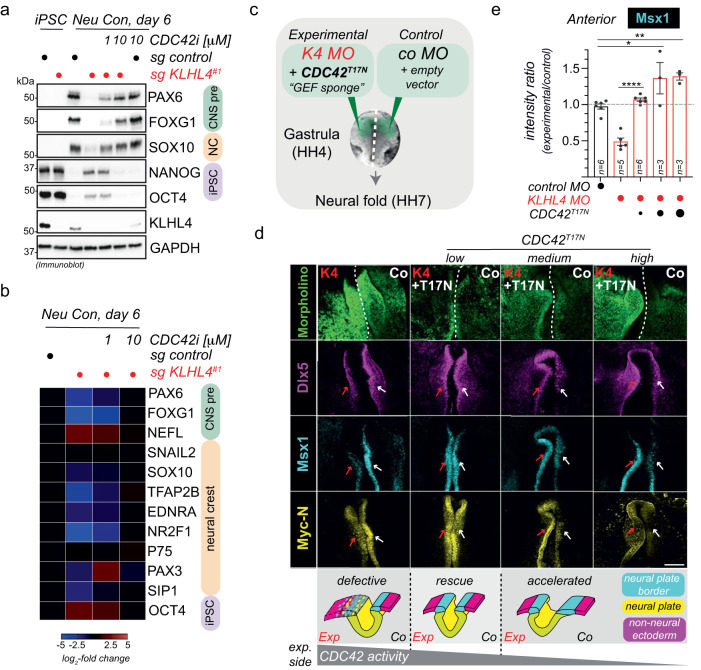
CRL3-KLHL4 balances CDC42 signaling to coordinate ectodermal patterning and neurulation in the vertebrate head. **a** CDC42 inhibition rescues neural conversion of KLHL4-deficient iPSCs. Control or KLHL4-depleted CRISPRi iPSCs were treated with DMSO or indicated concentrations of the CDC42 inhibitor ML141, subjected to neural conversion for 6d, and analyzed by immunoblot analysis for expression of CNS precursor (green), neural crest (orange), or pluripotency (purple) markers. GAPDH = loading control. *n* = 2 biological replicates. **b** CDC42 inhibition rescues neural conversion of KLHL4-deficient iPSCs. Control or KLHL4-depleted CRISPRi iPSCs were treated and subjected to neural conversion as described above and analyzed by qRT-PCR analysis for expression of lineage markers color-coded as above. Marker expression was normalized to control iPSCs. RPL27 = endogenous control. *n* = 2 biological replicates with three technical replicates each. **c** Experimental design for in vivo electroporations testing the ability of ^GFP^CDC42^T17N^, a constitutively inactive CDC42 variant that can bind GEFs but is deficient in recognizing effectors, to rescue KLHL4 MO (K4 MO) phenotype. Increasing concentrations of ^GFP^CDC42^T17N^ variant were co-expressed with K4 MO, while control MO (co MO) with empty vector was injected on the contralateral side. **d** Ectodermal patterning defect phenotype caused by loss of KLHL4 MO was rescued by co-expression of low dose CDC42^T17N^, but medium and high doses resulted in the appearance of accelerated neural folding as evaluated from whole-mount embryos after HCR FISH by using probes for *Msx1*, *Myc-N*, and *Dlx5*. Scale bar  = 250 µm. The cartoon depicts how an optimal amount of CDC42 activity is required for normal ectodermal patterning and neurulation. dashed line = midline of the embryo between the differentially treated sides. **e** Graph depicts ratios of anterior *Msx1* intensity of the experimental side relative to the control side of the embryo. For reference, *Msx1* intensity rations of embryos that were injected with control MO on both sides are shown. *n* = 3–5 embryos per condition as indicated by the number in the bar, error bars = s.e.m., K4 MO vs. CDC42^T17N^ low: *P* = 0.000044, Co MO vs. CDC42^T17N^ medium: *P* = 0.0126, Co MO vs. CDC42^T17N^ high: *P* = 0.0078, one-way ANOVA).

The above results suggested that loss of CRL3-KLHL4-dependent PAK1 ubiquitylation results in abnormally increased CDC42 activation. We therefore tested whether the CDC42 small molecule inhibitor, ML141 (CDC42i)^69^ would ameliorate ectodermal differentiation phenotypes in iPSCs lacking CRL3-KLHL4 activity. Intriguingly, while KLHL4-depleted iPSCs failed to undergo efficient neural conversion when cultured in the presence of the vehicle control DMSO, the addition of ML141 to the media rescued the aberrant differentiation program in a dose-dependent manner (Fig. 6a, b and Supplementary Fig. 13a). To expand these observations to our in vivo model, we next asked whether a constitutively inactive version of CDC42 still capable of GEF binding (CDC42^T17N^) could “sponge” excessive GEF activity and thus rescue KLHL4-depletion-induced phenotypes in the embryo (Fig. 6c). Strikingly, co-injection of low concentrations of a plasmid encoding CDC42^T17N^ with KLHL4 MO re-established ectodermal patterning to similar levels of control MO-injected embryos as evaluated by the expression of the respective domain markers *Msx1, Dlx5* and *MycN* (Fig. 6d, e). Surprisingly, in these experiments, we noted that increasing the concentration of CDC42^T17N^ resulted in an over-advanced neural plate border with significantly increased levels of *Msx1* compared to the control side of the embryo (Fig. 6d). In addition, the whole-mount and cross-section images indicated an acceleration of the neural plate folding, an opposite phenotype of what is caused by loss of KLHL4 (Fig. 6d, e and Supplementary Fig. 13b). These results suggest that expression of CDC42^T17N^ above a certain threshold reduces CDC42 activity below physiological levels, again resulting in aberrant patterning of ectodermal domains as well as an increased folding rate of the neural tube. To further substantiate this notion, we next performed CDC42^T17N^ injections in embryos without KLHL4 MO knockdown (Supplementary Fig. 13c). Also, under these conditions, CDC42^T17N^ expression caused a more defined neural plate border and increased *Msx1* expression as compared to the control side of the embryo, indicating an acceleration of the ectodermal patterning process (Supplementary Fig. 13d, e). Taken together, we conclude that post gastrulation, CRL3-KLHL4-dependent ubiquitylation of PAK1 in the anterior ectoderm is required to establish a precise level of CDC42 signaling. This ubiquitin-dependent regulation coordinates faithful ectodermal patterning and neural tube formation and both, too much or too little anterior CDC42 activity, results in aberrant ectodermal development.

## Discussion

**Fig. 7:**
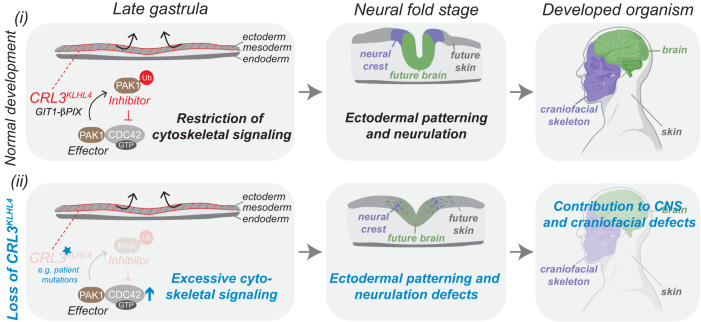
Modulation of CDC42 signaling by a ubiquitin-based effector-to-inhibitor switch coordinates ectodermal patterning and neurulation in the vertebrate head. Model depicting how CRL3-KLHL4-mediated restriction of anterior CDC42 signaling regulates ectodermal patterning. Canonically, CDC42 is activated by specific GEFs, including the GIT1-β-PIX complex, and inactivated by dedicated GAPs. The activated, GTP-bound form of CDC42 can bind and stimulate effector proteins, including PAK1, to mediate cytoskeletal and cell polarity-based downstream signaling. (i) During normal development, tissue-specific expression of CRL3-KLHL4 allows restriction of these canonical CDC42 signaling pathways in the anterior ectoderm of the gastrula. This inhibition relies on the CDC42-activating complex GIT1-β-PIX, which recruits CRL3-KLHL4 to its substrate PAK1. Ubiquitylation converts this canonical CDC42 effector kinases into a CDC42 inhibitor to restrict downstream signaling. This negative regulation ensures proper spatial ectodermal cell-fate specification, neural plate folding, and ultimately faithful development of the skin, the nervous system, and the craniofacial complex. (ii) Loss of CRL3-KLHL4 activity results in aberrant development. Hypomorphic mutations in *CUL3* or *KLHL4* impair CRL3-KLHL4 complex assembly and PAK1 ubiquitylation. This causes reduction of the negative feedback system and excessive CDC42 downstream signaling, which compromises ectodermal patterning, neural plate folding, and contributes to central nervous system and craniofacial phenotypes observed in patients with *CUL3* and *KLHL4* variants. Model was in part created using BioRender.com.

Differentiation processes are often driven by epigenetic, transcriptional, and translational network changes^35,70^. Here, we identify an essential posttranslational mechanism that regulates small GTPase signaling by converting a canonical pathway effector into an inhibitor to orchestrate key steps of early human development, a concept we hypothesize to be a common principle of regulating organogenesis. Specifically, we propose that (1) tissue-specific expression of the substrate adaptor protein KLHL4 spatiotemporally restricts monoubiquitylation activity of a CRL3 complex to the ectoderm layer and (2) monoubiquitylation by CRL3-KLHL4 balances cytoskeletal-based signaling by the ubiquitous small GTPase CDC42 and thereby coordinates ectodermal patterning and neurulation in the developing head (Fig. 7). Our work thus reveals an important function for ubiquitylation in coordinating cell-fate changes and morphological rearrangements during development and defines sensitive monitoring of CDC42 activity by a ubiquitin-dependent rheostat as crucial to establishing the anterior nervous system and craniofacial skeleton.

By using a combination of multiple lines of biochemical, cellular, and in vivo approaches, we show that tissue-specific expression of the CRL3-substrate adaptor KLHL4 implements monoubiquitylation-based restriction of CDC42 signaling pathways to coordinate ectodermal patterning and neurulation. Intriguingly, monoubiquitylation mediated by different CRL3-substrate adaptors is also required for other steps of neural crest development during specification (via KBTBD8^35,71^) and later during collagen secretion in cranial chondrocytes (via KLHL12^34^). We hence propose that CRL3-mediated monoubiquitylation of spatiotemporally regulated substrates is a common regulatory principle driving cell-fate determination and tissue morphogenesis that allows integration of multiple steps of the formation of the vertebrate head and possibly in the entire embryo.

Our results demonstrate that CRL3-KLHL4 is essential for early steps of brain and face formation, as we find previously identified neurodevelopmental and craniofacial disease-causing variants in *CUL3*^41,72,73^ to reduce CRL3-KLHL4 complex formation (Fig. 1a, b and Supplementary Fig. 1). In addition, a novel variant in *KLHL4* (p.I287V) in a patient with severe intellectual disability, brain malformations, and craniofacial defects, was not able to support ectodermal differentiation in vitro (Fig. 1e and Supplementary Fig. 3) or in vivo (Fig. 1f–h*)*. Although this patient was recently diagnosed with VPS35L-associated Ritscher–Schinzel syndrome (OMIM: #618981) based on a previously unrecognized homozygous *VPS35L* variant^74^, the patient presents on the severe end of the spectrum of *VPS35L-*associated phenotypes^75^ and other genetic factors such as the hypomorphic KLHL4 (p.I287V) variant characterized in this manuscript might therefore contribute to the severity of the phenotype. Our model is further corroborated by previous reports that demonstrate hyperactivating variants in PAK1 and CDC42 to result in various neural and neural crest-derived deficiencies in patients, including diverse neurodevelopmental and craniofacial defects^63,76^. Thus, an optimal amount of CDC42 activity is crucial for cranial morphogenesis.

CDC42 has been shown to control diverse aspects of early embryonic development by controlling actomyosin organization and cell polarity establishment^68,77–85^. We find that CRL3-KLHL4 regulates cell morphology and actin flow rates in vitro, and CDC42 inhibition rescued the ectodermal patterning and neural plate folding defect caused by a loss of KLHL4 in vivo (Fig. 6c, d*,* Supplementary Fig. 11a–k, and Supplementary Movie 1). Our data thus suggest that CDC42 restriction by CRL3-KLHL4-dependent PAK1 monoubiquitylation in the anterior ectoderm is a means to coordinate morphological rearrangements and cell-fate commitment through modulating actin cytoskeleton-associated cellular functions. Together with established roles of mechanical forces in directing embryonic stem cell behavior^86,87^, our study raises the exciting possibility that ubiquitin-dependent regulation of the actin cytoskeleton can be responsible for hard-wiring cell fates during development.

In cellular ubiquitylation assays, we find that CRL3-KLHL4 predominantly mediates the attachment of up to three ubiquitin moieties to the kinase domain of PAK1. Yet, mimicking monoubiquitylation by fusion of a single ubiquitin moiety to the C-terminus was sufficient to rescue KLHL4-depletion-induced aberrant CDC42 activation and defects in ectodermal differentiation. These findings raise intriguing questions regarding the mechanisms how CRL3-KLHL4 ensures multi-monoubiquitylation of PAK1, whether there are preferred sites of ubiquitin attachment, and whether there is a difference in downstream effects depending on the number of ubiquitins attached to PAK1. Future studies will be geared toward answering these questions to determine the molecular and structural basis of how ubiquitylation converts PAK1 into an inhibitor of CDC42.

Our mechanistic analyses reveal a previously unrecognized principle of small GTPase signaling that employs a complex enzymatic logic to coordinate ectodermal patterning and neurulation. Monoubiquitylation of the canonical CDC42 effector kinase PAK1^67,68^ results in the inhibition of CDC42. Intriguingly, CRL3-KLHL4 uses the CDC42-activating complex GIT1-β-PIX as co-adaptor to recruit and monoubiquitylate PAK1, which thus directly participates in the effector-to-inhibitor conversion. Recent reports have demonstrated that GIT-PIX complexes can form molecular condensates that concentrate limited quantities of enzymes to distinct cellular compartments, including focal adhesion complexes^56,88^. Therefore, assembling activators and effectors in such membraneless compartments and utilizing monoubiquitylation to convert effectors into inhibitors may serve as a rapid means to rewire CDC42-based cytoskeletal signaling pathways in response to morphogens. Given the prevalent roles of the Rho family of small GTPases during embryogenesis^83^, we predict that similar effector-to-inhibitor conversions will be a common mechanism to balance small GTPase signaling to coordinate tissue morphogenesis and cell-fate commitment during other aspects of development.

## Methods

### Plasmids, antibodies, proteins, and other key resources

Key resources used in this study are summarized in Supplementary Data 5.

### Mammalian cell culture and transfections

HEK293T and RPE-1 cells were maintained in DMEM with 10% fetal bovine serum. Plasmid transfections were carried out using PEI. siRNA transfections were carried out with Lipofectamine RNAiMAX (Invitrogen) according to the manufacturer’s instructions using 10 nM for each siRNA. Cells were routinely tested for mycoplasma using the MycoAlert Mycoplasma Detection Kit from Lonza (LT07-118).

### Pluripotent stem cell culture

iPSCs (WTC with dCas9-KRAB, AICS90) and hESCs (H1 line, WAe001-A) were maintained under feeder-free conditions on Matrigel-coated plates (#354277, BD Biosciences) in mTeSR^TM^1, (#05871/05852, StemCell Technologies Inc.). iPSCs were routinely passaged with acctuase (# 07920, StemCell Technologies Inc.), while hESCs were routinely passaged with collagenase (#07909, StemCell Technologies Inc.). Cells were routinely tested for mycoplasma using the MycoAlert Mycoplasma Detection Kit from Lonza (LT07-118).

### Lentiviral infections

Lentiviruses were produced in HEK293T cells by cotransfection of lentiviral constructs with third-generation packaging plasmids (Addgene) for 48–72 h. Transduction was carried out by infecting 2 × 10^5^ hES H1 cells per well of a six-well plate with lentiviruses in the presence of 6 μg/ml Polybrene (Sigma) and 10 μM Y-27632 ROCK inhibitor. For transduction of lentiviruses carrying ectopic expression vectors, cells were centrifuged at 1000 × *g* at 30 C for 90 min. Media was replaced with 2 mL mTESR1 containing 10 μM Y-27632 ROCK inhibitor. After 4–6d of selection with appropriate antibiotic (1 μg/ml puromycin for sgRNA constructs, 200 μg/ml G418 for pINDUCER20 constructs), iPSCs or hESCs were analyzed and used in differentiation experiments.

### Neuroectodermal differentiation of iPSCs

Neural conversion of iPSCs was performed using STEMdiff^TM^ Neural Induction Medium (#05831, StemCell Technologies Inc.) in combination with a monolayer culture method according to the manufacturer’s technical bulletin (#28044). In brief, single-cell suspensions were prepared by treatment of hES cells with accutase, and 1.5–2.0 × 10^6^ cells were seeded per well of a six-well plate in 4 mL STEMdiff^TM^ Neural Induction Medium supplemented with 10 μM Y-27632 ROCK inhibitor. Neural induction was performed for indicated time periods with daily medium change.

### iPSC rescue experiments

iPSCs (WTC with dCas9-KRAB, AICS90) were transduced with control sgRNAs or sgRNAs targeting KLHL4 and selected and maintained in 1 μg/ml puromycin. Cells were then stably transduced with pINDUCER20 plasmids (with indicated KLHL4^FLAG^ constructs containing wobble mutations that render them resistant to sgRNA recognition or indicated ^HA^PAK1 constructs. To mimic constitutively monoubiquitylated PAK1, Ub or UbI44A were fused to the C-terminus of PAK1 using a flexible 22 G–S linker). Cells were selected and maintained with 200 μg/ml G418 for 4-5d. For the rescue experiments, these cell lines were then treated with or without 1 μg/ml doxycycline to induce construct expression and subjected to neural conversion for indicated time periods. Cells were harvested for small GTPase pull-down assays, immunoblotting, RNA extraction, or fixed for immunofluorescence analysis.

### Differentiation of neural progenitor cells and neurons from embryoid bodies

Embryoid body (EB) formation from hESCs (H1 line) were performed using Aggrewell^TM^800 plates (#27865, StemCell Technologies Inc.) and APEL2 medium (#05270, StemCell Technologies Inc.) following the guidelines of the manufacturer’s technical manual (#29146). In brief, single-cell suspensions were prepared by treatment of hES cells with accutase (#07920, StemCell Technologies Inc.), and 1 × 10^6^ cells were seeded per well of an Aggrewell^TM^800 plate in APEL2 medium supplemented with 10 μM Y-27632 ROCK inhibitor (#72307, StemCell Technologies Inc.) followed by 72 h incubation with daily media change. Embryoid bodies were then embedded into Matrigel and differentiated in APEL2 medium for 6d. The medium was replaced every day.

### Immunoprecipitations

For co-immunoprecipitation experiments, HEK293T or RPE-1 cells were transiently transfected with indicated FLAG-tagged, MYC-tagged, and/or HA-tagged constructs and incubated for 48 h at 37 **°**C with 5% CO_2_. Cells were harvested by scraping in 1×PBS and centrifuged at 300 × *g* for 5 min. The cell pellets were either stored at −80 **°**C or directly used for immunoprecipitation experiments. For each condition, typically 1 × 10-cm dishes (HEK293T) or 1× 15-cm dishes (RPE-1) were used. Cells were lysed in two pellet volumes of ice-cold lysis buffer (20 mM HEPES pH 7.3 containing 110 mM potassium acetate, 2 mM magnesium acetate, 50 mM NaCl, 1 mM EGTA, 0.1% NP-40, 1 × protease inhibitors (Roche), 1× Phos-Stop (Roche), and 2 mM phenanthroline. Cells were sonicated, and the lysates were cleared by centrifugation at 20,000 x g for 25 min. To remove residual lipids, the supernatant was filtered through a 0.22-μm filter (Millex-GV). Subsequently, the lysates were quantified using Pierce 660 nm reagent (Thermo, #22660), and equal amounts of lysates were incubated with ANTI-FLAG-M2 agarose (Sigma) for 2 h at 4 °C. Beads were then washed three times with lysis buffer and eluted in 2× urea sample buffer (150 mM Tris pH 6.5, 6 M urea, 6% SDS, 25% glycerol and a few grains of bromophenol blue) followed by immunoblot analysis for interaction partners.

For compPASS-based mass spectrometry, HEK293T cells transiently transfected with different KLHL4^FLAG^ constructs were lysed and subjected to anti-FLAG immunoprecipitation as described above (4 × 15-cm dishes per condition). Beads were then washed three times with lysis buffer and eluted in lysis buffer supplemented with 0.5 mg/mL 3xFLAG peptide (Sigma). Eluted proteins were precipitated by adding 20% TCA followed by overnight incubation on ice. Protein pellets were washed three times with ice-cold 90% acetone in 0.01 M HCl, air-dried, and further processed for mass spectrometry analysis as described below.

For immunoprecipitation of endogenous KLHL4 complexes, lysates of 2 × 15-cm dishes of hESC (H1 line) were prepared as described above. After incubation with 20 μg anti-KLHL4 or control antibodies (mIgGs, Santa Cruz) at 4 °C for 1 h, Protein G beads (Roche) were added for 2 h. After washing with lysis buffer, bound proteins were eluted with 2× SDS sample buffer and analyzed by immunoblotting.

For immunoprecipitation of ^FLAG^CUL3 complexes from hESC undergoing early stages of ectodermal differentiation, hESCs (H1 line) stably transduced with ^FLAG^CUL3 or indicated congenital disease-causing variants were subjected to neural conversion for 1d and harvested (2 × 15-cm dishes per condition). Cell pellets were lysed in two pellet volumes of lysis buffer (20 mM HEPES pH 7.3, 150 mM NaCl, 110 mM KOAc, 2 mM Mg(OAc)_2_, 5 mM EDTA, 5 mM EGTA, 0.2% NP-40) supplemented with 2 mM phenantroline and protease inhibitors (Roche) on ice followed by brief low-amplitude sonication and subsequent trituration through 25 gauge needles. Lysates were cleared by centrifugation, passaged through a 0.45 μm membrane filter, incubated with Protein G agarose for 30 min at 4 °C to remove nonspecific interactors, and incubated with ANTI-FLAG-M2 agarose (Sigma) for 1 h at 4 °C. After washing with lysis buffer, FLAG-tagged protein complexes were eluted with lysis buffer containing 0.5 mg/mL 3xFLAG peptide in three 15 min incubations at 30 °C, 800 rpm. Eluted proteins were precipitated by adding 20% TCA followed by overnight incubation on ice. Protein pellets were washed three times with ice-cold 90% acetone in 0.01 M HCl, air-dried, and further processed for mass spectrometry analysis as described below.

### Mass spectrometry analysis

Eluates from FLAG IPs were precipitated with TCA overnight, reduced, alkylated, separated from FLAG peptide via S-Trap^TM^ mini columns (Protifi), and in-column digested with trypsin overnight. Tryptic digests were analyzed using an orbitrap Fusion Lumos tribrid mass spectrometer interfaced to an UltiMate3000 RSLC nano HPLC system (Thermo Scientific) using data-dependent acquisition. Specifically, peptides were loaded with autosampler and trapped in an Acclaim PepMap 100 trap column (75 µm × 2 cm, Thermo Scientific) at 4 µL/min with 100% solvent A (0.1% formic acid in water) for 5 min. Peptides were then separated with an Acclaim PepMap RSLC column (75 µm × 25 cm, C18 2 µm 100 Å, Thermo Scientific) at 0.3 µL/min with a gradient of 4–31% solvent B (80% ACN, 0.1% formic acid) in 140 min followed by a 20 min gradient to 50% B. The column was then washed with 99%B for 5 min before returning to 1% B. Column was equilibrated at 1% B for 10 min before the next injection. The column was maintained at room temperature. Mass spectrometry data were recorded between 8 and 175 min using data-dependent acquisition with a cycle time of 1 s. Full-scan MS1 was acquired in the orbitrap with resolution 240,000 (*m/z* 200). HCD MS/MS spectra was acquired in the linear ion trap at unit mass resolution with isolation window 1.2 *m/z* using turbo scan. HCD energy was 30%. AGC was 250% for MS1 and 150% for MS2. Precursors with charges between 2–7 was selected for MS2, Dynamic exclusion was set a ± 10 ppm for 60 s with isotopes excluded. Initial protein identification was carried out using Proteome Discoverer (V2.4) software (Thermo Scientific) against the uniprot human protein database (v2020.10.29) with full trypsin digestion and up to four missed cleavages. Precursor mass tolerance was 10 ppm and fragment mass tolerance was 0.6 Da. M oxidation, K ubiquitinylation (GG), and STY phosphorylation, as well as protein N-terminal acetylation and ubiquitinylation (GG) were set as variable modifications. Cysteine modification by MMTS ( + 45.988) was set as fixed modification. Percolator FDR control was set at 1% for both protein and peptide ID with concatenated target/decoy database. Proteins with 1 peptide ID was included in the report. To compare the impact of disease-causing variants on known CUL3 interactors, the average intensity of the top three peptides of each interactor was normalized against that of CUL3 for each condition. Normalized known CUL3 interactors were compared to WT CUL3 (set to 1) and plotted as a heatmap. For each CUL3 IP condition, two biological replicates were performed and averaged. To determine high-confidence interaction partners for KLHL4, search results from Proteome Discoverer of KLHL4^FLAG^ IPs were exported into Scaffold4 and compared with ∼30 reference immunoprecipitations against different FLAG-tagged bait proteins using a python script programmed according to the CompPASS software suite^50^. For the determination of the KLHL4 interaction network, three independent KLHL4^FLAG^ IPs were compared as replicates against the reference IPs. Thresholds for high-confidence interaction partners (HCIPs) were top 5% of interactors with the highest Z-score and highest WD score. To narrow down putative substrates of KLHL4 in the interaction map, we compared relative total spectral counts for each HCIP found in WT KLHL4 IPs to the ones found in one replicate of KLHL4 I287V and KLHL4ΔCUL3 IPs.

### Cellular ubiquitylation assays

To detect ubiquitylation of candidate substrates of KLHL4, RPE-1 cells were transiently transfected with ^HIS^Ubiquitin WT or chain formation-deficient ^HIS^Ubiquitin (K^0^) and indicated epitope-tagged components of the GIT-PIX-PAK module in the absence or presence of KLHL4^FLAG^ variants. Cells were harvested 48 h after transfection, washed with PBS, lysed in 8 M urea, 50 mM sodium phosphate, pH 8.0, and sonicated. His-Ubiquitin conjugates were purified using Ni-NTA agarose (Qiagen). Modified proteins were detected by immunoblotting using the indicated antibodies.

### Mapping of ubiquitylation sites in PAK1

To detect ubiquitylation in PAK1, 20x 15 cm of RPE-1 cells were transiently transfected with ^HIS^Ubiquitin, ^HA^PAK1, and KLHL4^FLAG^. Cells were harvested 48 h after transfection, washed with PBS, lysed in in 8 M urea, 50 mM sodium phosphate, pH 8.0 and sonicated. His-Ubiquitin conjugates were purified using Ni-NTA agarose (Qiagen), washed and eluted with in 8 M urea, 50 mM sodium phosphate, 200 mM imidazole pH 8.0. Eluates were diluted to 1 M urea with lysis buffer (20 mM HEPES pH 7.3 containing 110 mM potassium acetate, 2 mM magnesium acetate, 50 mM NaCl, 1 mM EGTA, 0.1% NP-40) and subjected to anti-HA immunoprecipitation. After washing, proteins were eluted from HA beads with 5% SDS, reduced with DTT, alkylated with MMTS, and in-column digested with trypsin on S-TrapTM mini columns (Protifi) overnight. Eluted peptides were first analyzed by nanoLC-MS/MS with using the same data-dependent method. For better site localization, the sample was analyzed a 2nd time where ubiquitylated peptides identified in the 1st analysis were specifically isolated and fragmented in a parallel reaction monitoring experiment. The resulting data were searched with Proteomics Discoverer (v2.5), and sites of ubiquitylation were manually verified.

### Purification of recombinant proteins

^His^MBP, ^His^MBP-GIT1^WT^, ^His^MBP-GIT1^RK3D^, GST, GST-RBD, and GST-PBD were purified from Rosetta II (DE3) competent cells using previously established protocols^89^. In brief, cells were grown at 37 °C until an OD_600nm_ = 1.5–2.0 was obtained, chilled, and protein expression was induced with 500 nM IPTG at 16 °C for 16 h. Bacteria were harvested, resuspended in lysis buffer (0.1 M Tris [pH 8.0], 0.5 M NaCl, 5 mM EDTA, 0.1% Triton-X100) supplemented with a protease inhibitor cocktail (Roche), and lyzed in a chilled LM10 microfluidizer at 15,000 psi. Lysates were clarified by centrifugation at 4 °C, 50,000 × *g* for 30 min and incubated with Ni-NTA agarose (Qiagen) or glutathione Sepharose (GE Healthcare) at 4 °C for 2 h. For ^HisMBP^GIT1^WT^ and ^HisMBP^GIT1^RK3D^, beads were washed with 10–15 bead volumes of wash buffer (0.1 M Tris [pH 8.0], 0.5 M NaCl, 20 mM Imidazole), and subsequently eluted with 5–10 bead volumes of elution buffer (0.1 M Tris [pH 8.0], 0.5 M NaCl, 300 mM Imidazole). For GST-tagged proteins, beads were washed with 10–15 bead volumes of wash buffer (0.1 M Tris [pH 8.0], 0.5 M NaCl), and subsequently eluted with 5–10 bead volumes elution buffer (0.1 M Tris [pH 8.0], 0.5 M NaCl, 20 mM Glutathione). Protein eluates were dialyzed overnight at 4 °C in dialysis buffer (0.1 M Tris [pH 8.0], 0.3 M NaCl) and concentrated using amicon ultra concentrators, MWCO 30 kDa (Millipore Sigma) and further purified via gel filtration proteins using a Superdex 200 Increase (GL 10/300) column. Proteins were concentrated, aliquoted, and snap-frozen in liquid N_2_ to be stored at −80 °C.

KLHL4^FLAG^ was purified from HEK293T cells (10 × 15-cm dishes). Lysates were prepared and subjected to anti-FLAG immunoprecipitation as described above. To remove endogenous interaction partners, beads were washed three times with lysis buffer containing 1 M NaCl, three times with lysis buffer containing 2% NP-40, and three times with lysis buffer, followed by elution from the beads with lysis buffer containing 3×FLAG peptide (0.5 mg/ml). KLHL4 protein was quantified against BSA using coomassie-stained SDS page gels.

### In vitro reconstitution of GIT1-KLHL4 binding

To reconstitute GIT1-KLHL4 binding in vitro, 1.2 mM ^His^MBP, ^His^MBP-GIT1^WT^, and ^His^MBP-GIT1^RK3D^ were immobilized on amylose beads TB buffer (20 mM HEPES [pH 7.3], 100 mM KC_2_H_3_O_2_, 2 mM Mg(C_2_H_3_O_2_)_2_ 1 mM EGTA, 0.1% NP-40) for 30 min at 4 °C. Excess/unbound GIT1 was removed by washing once with TB buffer. 0.6 mM KLHL4^FLAG^ were added in total binding volume of 200 mL an incubated for 1 h at 4 °C, followed by three washes with TB buffer, elution in 2× urea sample buffer, and immunoblot analysis.

### Small GTPase pull-down assays

For determining the amount of GTP-bound small GTPases, iPSCs subjected to neural conversion for 3d (1 × 10-cm dises per condition) were washed with PBS, followed by lysis in 250 mL lysis buffer (50 mM Tris pH 7.5, 10 mM MgCl_2_, 0.5 M NaCl, and 2% Igepal) on ice. Lysates were sonicated, cleared by centrifugation at 4 °C at full speed in a tabletop centrifuge for 10 min, and quantified via Pierce 660 nm protein assay. Equalized lysates were incubated with GST, GST-RBD, and GST-PBD immobilized on glutathione agarose for 1 h at 4 °C (20 mL of beads at a 2 mg/mL concentration). Beads were washed once with wash buffer (25 mM Tris pH 7.5, 30 mM MgCl_2_, 40 mM NaCl) and taken up in 2× urea sample buffer, followed by immunoblot analysis. Relative small GTPase activity was calculated by dividing the GTP-bound state present in the GST pull-down fraction to the total levels of each small GTPase detected in the input fractions followed by normalization to control cells.

### Quantitative real-time PCR (qRT-PCR) analysis

For qRT-PCR analysis, total RNA was extracted and purified from cells using the NucleoSpin RNA kit (#740955, Macherey Nagel) and transcribed into cDNA using the SuperScript™ IV First-Strand Synthesis System (#18091050, ThermoFisher Scientific). Gene expression was quantified by PowerUp SYBR Green qPCR (#A25741, ThermoFisher Scientific) on a CFX96 Real-Time System (Bio-Rad). Nonspecific signals caused by primer dimers were excluded by dissociation curve analysis and the use of nontemplate controls. Loaded cDNA was normalized using RPL27 as an endogenous control. Gene-specific primers for qRT-PCR were designed by using NCBI Primer-Blast. Primer sequences can be found in Supplementary Data 5.

### Cluster analysis

mRNA abundance was measured by RT-qPCR for different conditions. The datasets were plotted as a heatmap in Python using the Seaborn library. Hierarchical clustering of samples was performed using the Bray-Curtis method with average linkage.

### Immunofluorescence microscopy

For immunofluorescence analysis, self-renewing or differentiated iPSCs or hESCs were seeded on Matrigel-coated coverslips using accutase, fixed with 4% formaldehyde in PBS for 20 min, permeabilized with 0.5% Triton in PBS for 10 min, blocked in 2% BSA for 1 h and stained with indicated primary and secondary antibodies and/or Hoechst 33342 for 1 h. Images were taken using a Nikon A1R + HD confocal microscope system (Nikon Instruments, Melville, NY). 488 nm, 561 nm, and 640-nm laser lines provided illumination for hoechst, AF 488, Rhodamine Red X and AF647 fluorophores, respectively. Data were acquired using Galvano mode at 1024 × 1024 with no line averaging A Z-piezo stage (Physik Instrumente USA, Auburn, MA) allowed for rapid imaging in Z every 1 µm over an 8-µm Z distance. NIS-Elements (Nikon, Melville, NY) controlled all equipment. All images were maximum intensity projections and processed using ImageJ/FIJI.

### Microinjections to chick embryos

The chick embryo electroporations were performed as previously described^90^. Briefly, Hamburger Hamilton (HH) stage 4 (gastrula) embryos were collected from fertilized chicken eggs on punched Whatman filter papers and placed in an electrode chamber filled with Ringer’s Solution for double-sided electroporations. A glass needle pipette was filled with either the translation-blocking FITC (fluorescein isothiocyanate)-conjugated Morpholino or Control Morpholino at a concentration of 1.5 mM with 1 μg/μL carrier DNA, or alternatively, with an experimental plasmid with the respective empty vector for the control side. The regents were marked with two different food colors to make sure the injected area did not cross over the midline of the embryo. After the double injection, a complementary electrode was gently placed above the embryo in Ringer’s to apply current to deliver the material into the ectodermal germ layer. Embryos were then removed and placed into a dish with albumin to grow at 38 °C and collected at HH7-8. The embryos were then checked for fluorescence and fixed in 4% PFA–PBS–0.2% Tween for 1.5 h at room temperature or overnight at 4 °C, and dehydrated by using a gradient to bring them to methanol where they were stored in −80 °C.

### In situ hybridizations

Fluorescent in situ hybridization by using hybridization chain reaction (FISH-HCR). The FISH-HCR was performed as previously described^91^ with the following modifications: (1) After applying the probes overnight, the embryos were washed for 3 h, changing the wash buffer each hour. (2) After applying the hairpins overnight, the embryos were washed for 2 h, changing 5 × SSCT each hour, and DAPI was applied for 30 min before the final 30 min wash. (3) After the hairpins were washed, embryos were postfixed with 4% PFA for 15 min at room temperature and washed in 0.2% PBST 2 × 10 min. The embryos were then imaged as whole mounts.

### Chromogenic in situ hybridization

Whole-mount in situ hybridization was performed as described. Briefly, the KLHL4 probe was made by cloning the respective gene (bp 1887–2672, XM_420250.8) to a DNA vector from RT-PCR products made by using chicken whole embryo cDNA as a template. The digoxigenin-conjugated RNA probes were visualized by using anti dig-AP antibody and NCB/BCIP and postfixed with 4% FA for 1H RT.

### Whole-mount immunohistochemistry on chick embryos

Immunostainings were performed as previously described^46^. The embryos were washed with PBS and fixed with 4% PFA for overnight at 4 °C t room and washed in 0.2% PBST 3×20 min and blocked with 5% donkey/5% goat serum 0.2% PBST for 1 h RT. Pax7 primary antibody was diluted into the blocking buffer (1/10) and nutated in slow motion for 2days at 4 °C, washed 5 × 30 min RT and incubated with the Alexa secondary antibody (1/1000) overnight at 4 °C, washed 5 × 30 min RT and mounted for imaging by using the slow-fade mounting medium (Invitrogen ProLong^TM^ Gold Antifade Mountant).

### Cryosectioning

For cryosections, the embryos were rehydrated to 0.2%–PBST and incubated through a sucrose gradient (5% 15 min RT, 15% 2–3 h RT before incubation in 7.5% gelatin at 37 °C for 5–7 h) followed by embedding in cryomolds. Solidified gelatin blocks were flash-frozen in liquid nitrogen and stored at −80 °C until sectioned into 20-mm sections.

### IHC imaging

Imaging of the whole embryo and sections was carried out using an Andor Dragonfly 200 spinning disk confocal system coupled to a Zeiss AxioObserver (Zeiss, Thornwood, NY). A 20× apochromat air (NA 0.80) and 40× LWD apochromat water (NA 1.15) objectives were used for whole-mount and cross-sectional imaging, respectively. The samples were mounted in Prolong Grid antifade mounting medium. Andor integrated laser engine provided the excitation light using 405 nm (100 mW), 488 nm (150 mW), 561 nm (100 mW), 594 nm (100 mW), 640 nm (140 mW), and 730 nm (30 mW) laser lines, and using suitable emission wavelengths for each fluorophore. A Photometrics Prime 95B CMOS (Photometrics, AZ) camera was used in 12-bit mode with 3× gain. Illumination times and relative laser intensity were varied based on sample/fluorophore brightness. Exposure times and relative laser intensity was varied based on sample/fluorophore brightness. A Z-piezo stage (ASI Imaging, Eugene, OR) allowed rapid imaging in Z. Images were collected every 10 µm over 220 µm distance or every 1 µm over 40 µm distance for whole mount and cross-sections, respectively. All components were controlled by Micro-manager version 1.4.22 and were programmed by ADD. Tiled images were stitched together using the Grid/Collection stitching plugin^92^.

### Live-cell imaging

The day prior to imaging RPE-1 cells expressing inducible EGFP P-tractin or Td-tomato P-tractin were plated as single cells or to confluence for wound assays and treated with doxycycline (dox). The following day, cells were treated with imaging media containing Fluorobrite DMEM, 10% FBS, 100 U/ml pen/strep, 2 mM l-glutamine, 1:100 ratio of Oxyfluor and 10 mM DL-lactate to reduce photobleaching and phototoxicity. In some cases 1 µM SpyDNA 650 was added to the imaging media and washout after 1 hour to visualize cell nuclei. Single-cell imaging was carried out with a Yokogawa CSU-X1 confocal scanner attached to an automated Nikon Ti2 inverted microscope (Nikon, Melville, NY) using either a 60× CFI SR Plan Apo 60× water objective (NA 1.27) or a CFI Plan apo Lambda S 100XC silicone oil objective (NA 1.35). A Lun-X laser launch provided excitation for 405 nm (100 mW), 445 nm (70 mW), 488 nm (150 mW), 514 nm (150 mW), 561 nm (100 mW), and 640 nm (140 mW) laser lines, using suitable emission wavelengths for each fluorophore. A Photometric Prime 95B CMOS camera was used in 12-bit mode with 3× gain. A Piezo stage (PI) stage was used to rapidly capture Z-stacks every 0.5 µm over a 5–8-µm Z distance. An environmental chamber surrounding the microscope maintained cells at a constant 37 °C, with 10% CO_2_ and ~50% humidity (Okolabs, Tokyo). For determination of retrograde flow, images were acquired every 10 s. Maximum intensity projections were generated and were used for actin flow quantification. For all wound assays, a CFI Plan apo Lambda S 25XC silicone objective was used.

Alternatively, for wound assays, a Nikon A1R HD MP system was used (Nikon Instruments). In all, 488 nm (0.5–1.5%), 561 nm (1–2%), and 640 nm (3–5%) laser lines provided illumination for EGFP-P-tractin, td-tomato-P-tractin, and SpyDNA 650, respectively. Data were acquired using resonant mode and bidirectional scanning at 1024×1024 with 2× line averaging (frame rate = 7 frames/s), and image tiling in either 3  × 3 or 4 × 3 arrays. A Z-piezo stage (Queensgate) allowed for rapid imaging in Z every 1 µm over an 8-µm Z distance. Control and knockdown cells were imaged in adjacent chambers of an Ibidi eight-well chamber (Ibidi USA, WI) and data were acquired every 15 min for 12–16 h. Wounds were created using a sterile 200-µm pipet tip within 20 min of imaging. An environmental chamber surrounding the microscope maintained cells at a constant 37 °C, with 10% CO_2_ and ~50% humidity (Precision Plastics, Beltsville, MD). NIS-Elements (Nikon, Melville, NY) controlled all equipment on both microscopes.

### Image analysis and quantification

#### Kymograph analysis

A Fiji/ImageJ macro was created by ADD to generate three kymograph images, from which three slopes (distance over time) were quantified for each cell. 4D data (3D Z volumes over time) were the first maximum intensity projected (MIP) prior to kymograph generation. Data were converted into microns/sec.

#### Automated cell migration tracking

For tracking of cell migration in wound assays, the SpyDNA 650 channel was MIP, then automatically contrast adjusted and converted from 12-bit to 8-bit depth. The Fiji plugin trackmate (ref PMID: 27713081) was used to track nuclei. DoG detector (object diameter: 15, quality: 2) and simple LAP tracker (settings of 15, 15, 2) were used for tracking, and CSV files were exported and further analyzed in Microsoft Excel, where tracks below 15 data points were not included.

#### Whole-mount chick embryo and anterior cross-section analysis

All whole mounts and sections were displayed and analyzed using maximum intensity projections of Z-stacks. Measurements were done in Fiji by taking the average fluorescence intensity in manually drawn regions of interest. Measurements of the treated side were normalized to the control side within each embryo. For length measurements of overlapping regions in sections, manually drawn regions of interest were set as ROIs in Fiji before performing a conjunction operation to find the overlapping region. Tracing of neural tube shape was performed in Fiji by manual tracing as an ROI starting from the notochord before being converted to an X-Y coordinate for display and analysis. Calculation of the neural tube fold distance was done by setting the starting/notochord position as 0 in both X and Y coordinates before measuring the distance to the local maximum Y point.

#### Exome sequencing screen for BTB variants that affect CUL3 binding

To identify candidate disease-causing variants in BTB proteins, we searched exome sequencing data of patients with undiagnosed developmental diseases. We queried the public databases DECIPHER^93^ and denovo-db^94^ and also utilized genematcher^95^. We filtered for variants located in the BTB and BACK domain of the BTB protein. Hits were then tested for CUL3 binding by IP/IB experiments.

#### Human subjects

The patient carrying the *KLHL4* variant was consented for clinical and research-based exome sequencing as well as for research-based phenotyping through the University Medical Centre Utrecht, The Netherlands. Research with patient samples complied with all relevant ethical regulations of the University Medical Centre Utrecht, The Netherlands. A consent form to allow for publication was signed by the parents and made part of the medical record present at the University Medical Centre Utrecht, the Netherlands.

#### Quantification and statistical analysis

Immunoblot quantifications were performed using Fiji. Details of replicates for each experiment are provided in the figure legends. Statistical analyses were performed with GraphPad Prism v.9. Data are presented as mean ± s.e.m. unless otherwise noted in figure legend. For comparisons between two groups an unpaired *t* test was applied. For comparisons between three or more groups, a one-way ANOVA with Tukey’s multiple comparisons test was used.

### Reporting summary

Further information on research design is available in the Nature Portfolio Reporting Summary linked to this article.

## Supplementary information

Supplementary Information

Supplemental Dataset 1

Supplemental Dataset 2

Supplemental Dataset 3

Supplemental Dataset 4

Supplemental Dataset 5

Supplemental Movie 1

Reporting Summary

## Data Availability

All data supporting the findings are available in the main text, the supplementary material file, and the source data file. For identifying candidate disease-causing variants, we queried the public databases DECIPHER (https://www.deciphergenomics.org) and denovo-db (https://denovo-db.gs.washington.edu/denovo-db/) and also utilized genematcher (https://genematcher.org). To visualize the position of disease-associated CUL3 variants in the CUL3-BTB interface, we used the crystal structure of the KLHL11-CUL3 complex with the pdb entry 4APF. Proteomics data are provided in Supplementary Data 2 and 4 and were deposited into the Mass Spectrometry Interactive Virtual Environment (MassIVE) under the accession number MSV000090711. Requests for resources and reagents should be directed to and will be fulfilled by Achim Werner (achim.werner@nih.com).
