# Supplementary material for: A ubiquitin-based effector-to-inhibitor switch coordinates early brain, craniofacial, and skin development
Source: Nat Commun. 2023 Jul 26;14:4499. doi: 10.1038/s41467-023-40223-y (PMC10371987; doi:10.1038/s41467-023-40223-y)
Supplement: Supplementary file 1 — Supplementary Information [file 41467_2023_40223_MOESM1_ESM.pdf]

Supplemental Figures:

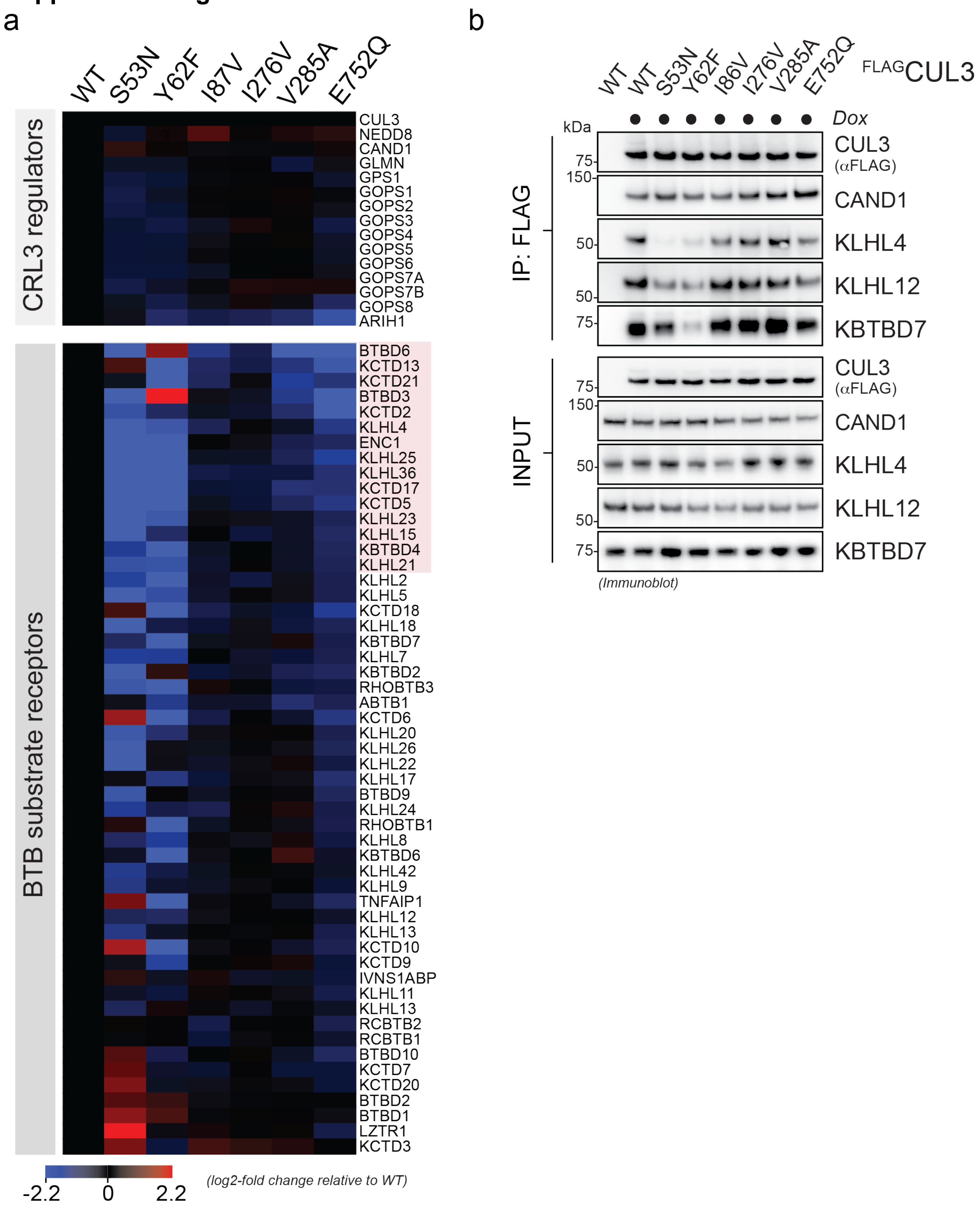

Supplemental Fig. 1: Neurodevelopmental and craniofacial disease-associated missense variants in CUL3 impact its interaction landscape during early stages of neuroectodermal differentiation. a) Neurodevelopmental and craniofacial disease-

causing variants in CUL3 change interactions with BTB substrate adaptors during early stages of ectodermal differentiation. FLAG-tagged (WT) or indicated CUL3 variant were affinity-purified from hESC (H1 line) undergoing 1d of neural conversion and analyzed by mass spectrometry. Heatmap depicts the relative abundance changes in known CUL3-interacting proteins relative to WT CUL3. Highlighted in red are the 15 BTB proteins that are 1.8-fold reduced in interaction in at least 3 different CUL3 variants. n = 2 biological replicates **b)** Verification of the mass spectrometry results for a subset of CUL3 interactors. FLAG-tagged (WT) or denoted CUL3 variant were affinity-purified from hESCs (H1 line) undergoing 1d of neural conversion and analyzed by immunoblotting with the indicated antibodies. Immunoblots are representative of 1 biological replicate.

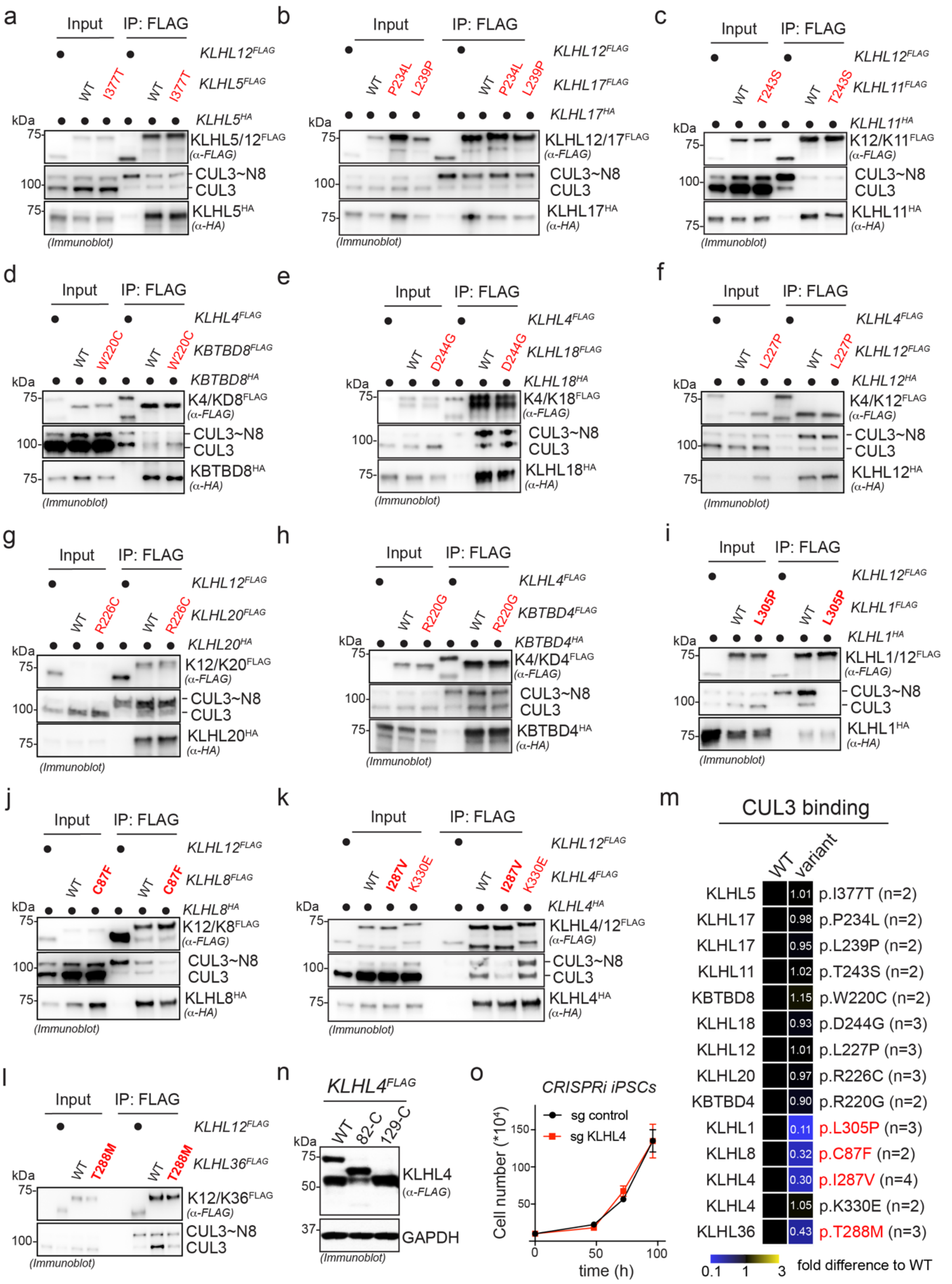

Supplemental Fig. 2: Biochemical screening of candidate disease-causing mutations from patients with undiagnosed diseases reveal several BTB variants that reduce CUL3 binding. a-l) Assessment of candidate-disease causing variants in

BTB proteins identified in patients with undiagnosed diseases on their impact on BTB homodimerization and CUL3 binding. HEK293T cells were transfected with FLAG-tagged WT or indicated BTB protein variants together with HA-tagged WT BTB protein followed by anti-FLAG immunoprecipitation and immunoblot analysis with indicated antibodies. For each panel, an unrelated FLAG-tagged BTB protein was used as specificity control for dimerization. Panels A-H depict BTB variants for which we did not detect obvious changes in CUL3 binding, while panels I-L depict BTB variants for which we detected a reproducible reduction in CUL3 binding. The number of replicates (n) for each immunoprecipitation experiment is indicated in Panel m. **m**) Heatmap depicting the fold difference in CUL3 binding for each candidate-disease causing BTB variant, revealing four variants (highlighted in red) that are reproducibly reduced in CUL3 binding. Values were quantified by measuring the total CUL3 signal over the FLAG signal and calculated relative to the respective WT BTB (which was set to 1). **n**) Ectopic expression of KLHL4^{FLAG} results in two isoforms in HEK293T cells. HEK293T cells were transfected with C-terminally FLAG-tagged WT KLHL4 and indicated N-terminal deletion constructs, lysed, and subjected to immunoblotting using anti-FLAG antibodies. GAPDH was used as loading control. M129 is the first methionine after M1 in the KLHL4 sequence and deletion of 128 amino acids from the N-terminus results in only the shorter isoform, while deletion of 82 amino acid still results in production of 2 isoforms. Thus, the two isoforms in WT KLHL4 likely originate from translation initiated from alternate start codons (M1 and M129). **o)** CRISPRi-mediated depletion of KLHL4 does not affect iPSC growth. Control of KLHL4-depleted iPSCs were counted at indicated times after seeding (mean of 3 biological replicates, -/+ s.d.).

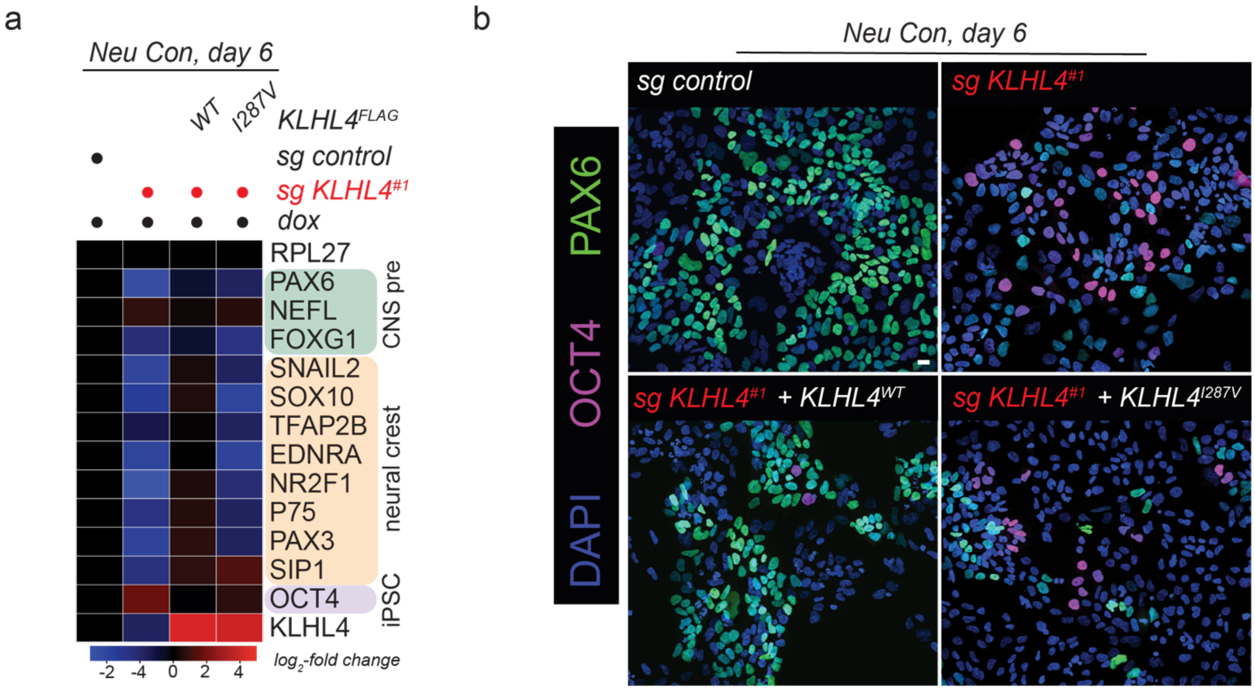

Supplemental Fig. 3: CRL3-KLHL4 is required for neural conversion. **a**) Control or KLHL4-depleted CRISPRi iPSCs stably expressing sgRNA-resistant and doxycycline-inducible wildtype (WT) or patient variant (I287V) $KLHL4^{FLAG}$ were generated followed by treatment with doxycycline (dox) and neural conversion for 6 days as indicated. Differentiation was monitored by qRT-PCR analysis for expression of indicated lineage markers (CNS precursors = green, neural crest = orange, iPSC = purple). Marker expression was normalized to control iPSCs. RPL27 = endogenous control**.** n=3 technical replicates. **b**) WT, but not the patient variant (I287V) KLHL4 can rescue neural conversion, as seen at the single cell level. Control or KLHL4-depleted CRISPRi iPSCs were reconstituted as described in panel A, treated with dox, and subjected to neural conversion for 8d, followed by immunofluorescence microscopy using antibodies against PAX6 (CNS precursor marker) and OCT4 (iPSC marker). Representative images of n= 2 biological replicates are shown. Scale Bar = 20µm.

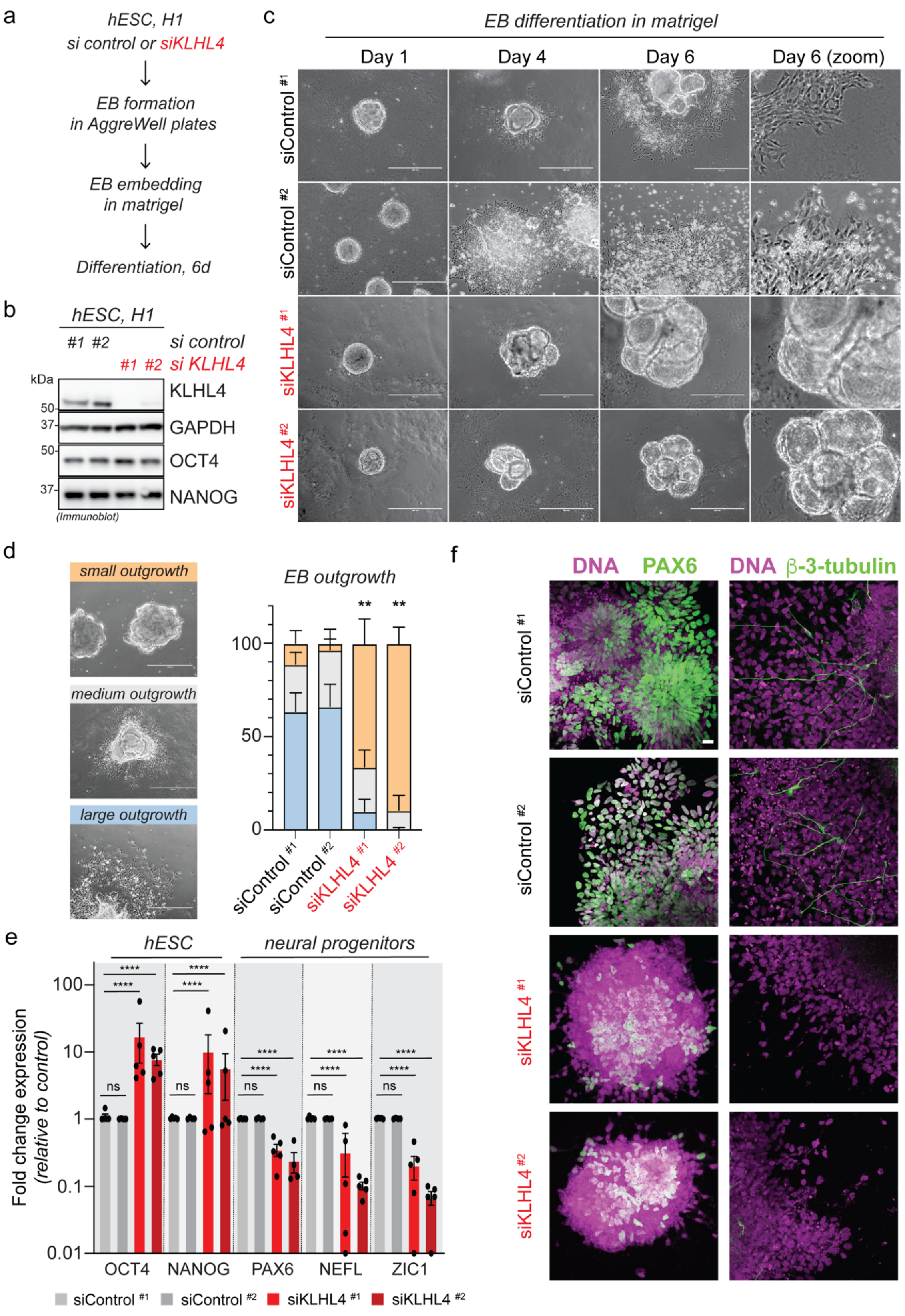

Supplemental Fig. 4: KLHL4 is required for formation of embryoid-body derived neural progenitor cells and neurons. a) Schematic overview of the embryoid body

differentiation paradigm used to differentiate hESCs into neural progenitors and neurons. **b**) KLHL4 depletion does not alter stem cell maintenance. hESCs (H1 line) were transfected with indicated control and KLHL4 siRNA for 72h, lysed, and subjected to immunoblotting using the antibodies against the pluripotency markers OCT4 and NANOG. GAPDH = loading control. Immunoblots are representative of 3 biological replicates.**c**) KLHL4 depletion does not interfere with embryoid body formation but reduces cell outgrowth during differentiation. Control or KLHL4-depleted embryoid bodies were generated, differentiated as described above, and phase contrast images were taken at indicated days of differentiation. Scale bar = 400μm. **d**) KLHL4 depletion significantly reduces large cell outgrowth during embryoid body differentiation. Bar graph depicts the relative percentage of embryoid bodies with small, medium, or large outgrowth detected in each condition. Representative images of embryoid bodies with different amount of cell outgrowth are shown on the left. n=7 for siControl$^{\#1}$, n=6 for siControl$^{\#2}$, n=11 for siKLHL4$^{\#1}$, n=4 for siKLHL4$^{\#2}$ with 10-50 embryoid bodies counted per biological replicate, error bars = s.e.m., KLHL4$^{\#1}$: $p = 0.00065$, KLHL4$^{\#2}$: $p = 0.00092$ unpaired t-test**.** Scale bar = 400μm **e**) KLHL4 depletion significantly reduces embryoid body-derived neural progenitor formation. Control or KLHL4-depleted embryoid bodies were differentiated for 6d as described above and subjected to qPCR analysis for the indicated hESC and neural progenitor markers. n= 5 biological replicates for siControl$^{\#1}$, siKLHL4$^{\#1}$, siKLHL4$^{\#2}$ and n = 4 for siControl$^{\#2}$, error bars = s.e.m, OCT4/NANOG/PAX6/NEFL/ZIC1: $p < 0.0001$ comparing siKLHL4$^{\#1}$ or siKLHL4$^{\#2}$ vs. siControl$^{\#1}$, one-way ANOVA**. f**) Control or KLHL4-depleted embryoid bodies were differentiated for 6d as described above and subjected to IF analysis using the neural progenitor marker PAX6 and the neuronal marker β-3-tubulin. Representative images of n= 3 biological replicates are shown. Scale bar = 40µm.

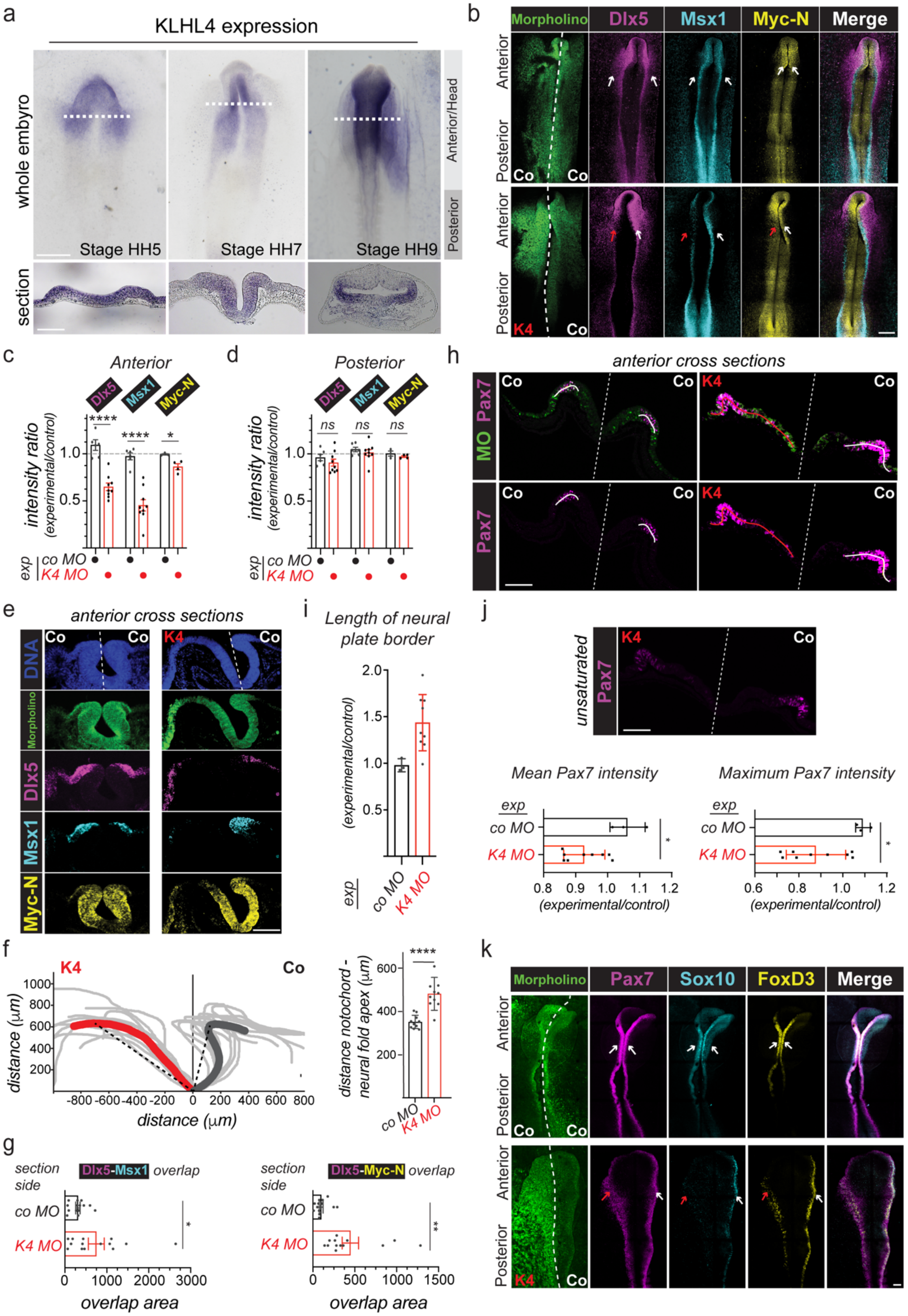

Supplemental Fig. 5: Anterior CRL3-KLHL4 ubiquitylation activity controls ectodermal patterning. a) In situ hybridization of *KLHL4* mRNA in the chicken embryo

(dotted lines mark the level of cross-section). **b**) K4 reduces anterior expression of *Dlx5*, *Msx1* and *Myc-N* shown by fluorescent in situ hybridization. Of note, the anterior part of the control embryo is also shown in Figure 1g. Scale Bar = whole mounts 250µm, sections 100 µm **c**) The ratio of fluorescence intensity between K4 and control side for *Dlx5*, *Msx1* (n=9 K4 embryos and 6 CoMo embryos, Dlx5: p=0.000036, Msx1: p=0.00002 unpaired t-test), and *Myc-N* (n=4 K4 embryos and 3 CoMo, p = 0.0163, unpaired t-test) at anterior axial levels. **d**) K4 shows no phenotype in the trunk (*Dlx5*, *Msx1*: n=9 K4 and 6 CoMo embryos, unpaired t-test; *Dlx5*, p= 0.4443; *Msx1* p= 0.4691; *Myc-N*: n=4 K4 and 3 CoMo embryos, unpaired t-test, *Myc-N*: p=0.2771). **e**) Midbrain region cross-sections from K4 and CoMo embryos (HH8-). Dotted line indicates the midline. Scale Bar = 100µm. **f**). The distance of the line from the notochord to the highest point (apex) of the neural fold is longer on the K4 side. Grey lines indicate individual, and thick lines average traces. Notochord was set to 0. (n=10, p=0.00091, unpaired t-test). **g**) The length of overlap between neighboring domains is increased on the K4 side (Dlx5-Msx1: K4 n=14, coMO n=12 sections, p = 0.0411, unpaired t-test; Dlx5-MycN: K4 n=13, CoMo n=13 sections, p = 0.002, unpaired t-test). **h**) Immunostaining of Pax7 demonstrates the length of the neural crest region as indicated by the red (K4) and white (CoMo) lines. **i**) The Pax7+ neural crest region is longer on the K4 side (1.43, n=9) as compared to the ratio between two control sides (0.97, n=3, p= 0.0017, unpaired t-test). Scale bar = 100 µm **j)** A non-saturated version of image in i shows reduced Pax7 intensity on K4 side, which is quantified in the graphs (K4 n= 9 CoMo n=3 embryos, unpaired t-test, mean intensity p=0.024, max intensity p=0.0014). Scale bar = 100 µm **k**) Expression of *Pax7*, *FoxD3* and *Sox10* during onset of cranial neural crest migration (n=3 K4 embryos and n=3 CoMo embryos). K4 = Morpholino mediated knockdown of KLHL4, CoMO = Control Morpholino. Scale Bar = 100µm

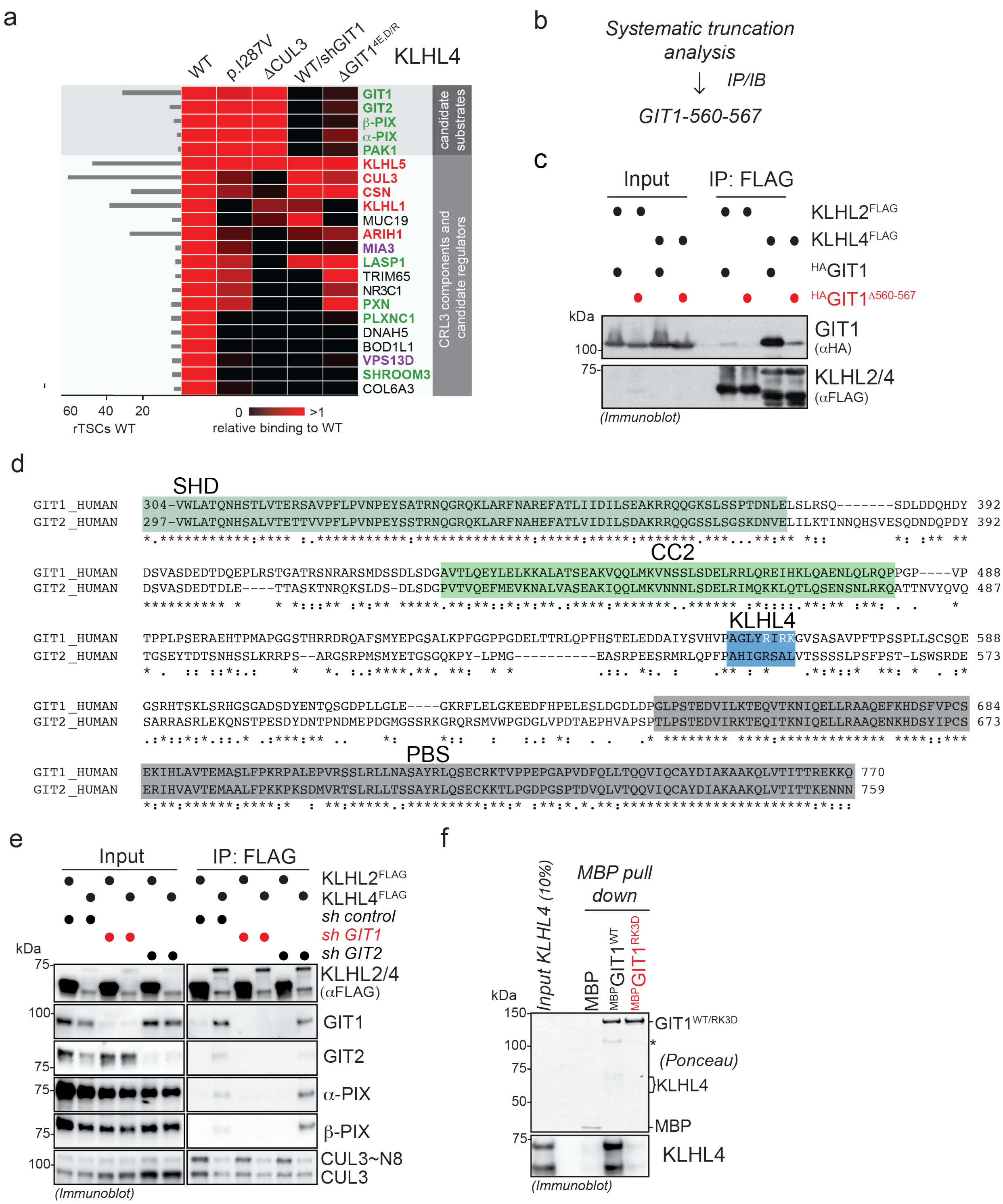

Supplemental Fig. 6: KLHL4 binds to GIT1 through a positively charged 6-amino acid stretch that is not conserved in GIT2. a) KLHL4 interacts with the GIT-PIX-PAK module through GIT1. FLAG-tagged wild-type KLHL4 (WT), patient variant KLHL4 (I287V), a severe CUL3-binding mutant of KLHL4 (ΔCUL3), and a GIT1-binding deficient mutant of KLHL4 (ΔGIT1), were affinity-purified from HEK293T cells. In

addition, FLAG-tagged wild-type KLHL4 (WT) was affinity-purified from HEK293T cells depleted of GIT1 (sh GIT1). Anti-FLAG IP fractions were analyzed for binding partners by mass spectrometry and CompPASS analysis. The heatmap depicts the relative binding of interactors identified for wild-type KLHL4 (n=3) in the respective KLHL4 variant (n=1). black = no interaction, red = equal or more interaction. The same heatmap for WT, I287V, and ΔCUL3 is also depicted in Fig 2B. **b**) GIT1 truncation analysis via IP/IB experiments from HEK293T cells reveals GIT1-560-567 as region for KLHL4 interaction. **c**) Amino acids 560-567 in GIT1 are required for interaction with KLHL4. HEK293T cells were co-transfected with FLAG-tagged WT KLHL4 and HA-tagged GIT1 or GIT1Δ560-567 lysed, and subjected to anti-FLAG IP followed by immunoblotting with indicated antibodies. KLHL2^{FLAG} was used as a specificity control for interaction. Immunoblots are representative of 2 biological replicates. **d**) Amino acids 560-567 in GIT1 are not conserved in its close homologue GIT2. Sequence alignment (generated using *clustal*) showing the C-terminal part of GIT1 and GIT2 with structural domains and KLHL4 interaction site highlighted in different colors. **e**) GIT2 is not required for binding of CRL3-KLHL4 to the GIT-PIX-PAK signaling module. Control, GIT1-depleted, or GIT2-depleted HEK293T cells were transfected with KLHL4^{FLAG} or KLHL2^{FLAG}, lysed, and subjected to anti-FLAG IPs followed by immunoblotting with indicated antibodies. Immunoblots are representative of 1 biological replicate. **f**) KLHL4 directly interacts with GIT1. KLHL4^{FLAG} purified from HEK293T cells under stringent conditions was incubated with recombinant MBP-tagged GIT1^{WT} or GIT1^{RK3D} and subjected to MBP pull down, followed by immunoblotting with indicated antibodies. Immunoblots are representative of 2 biological replicates.

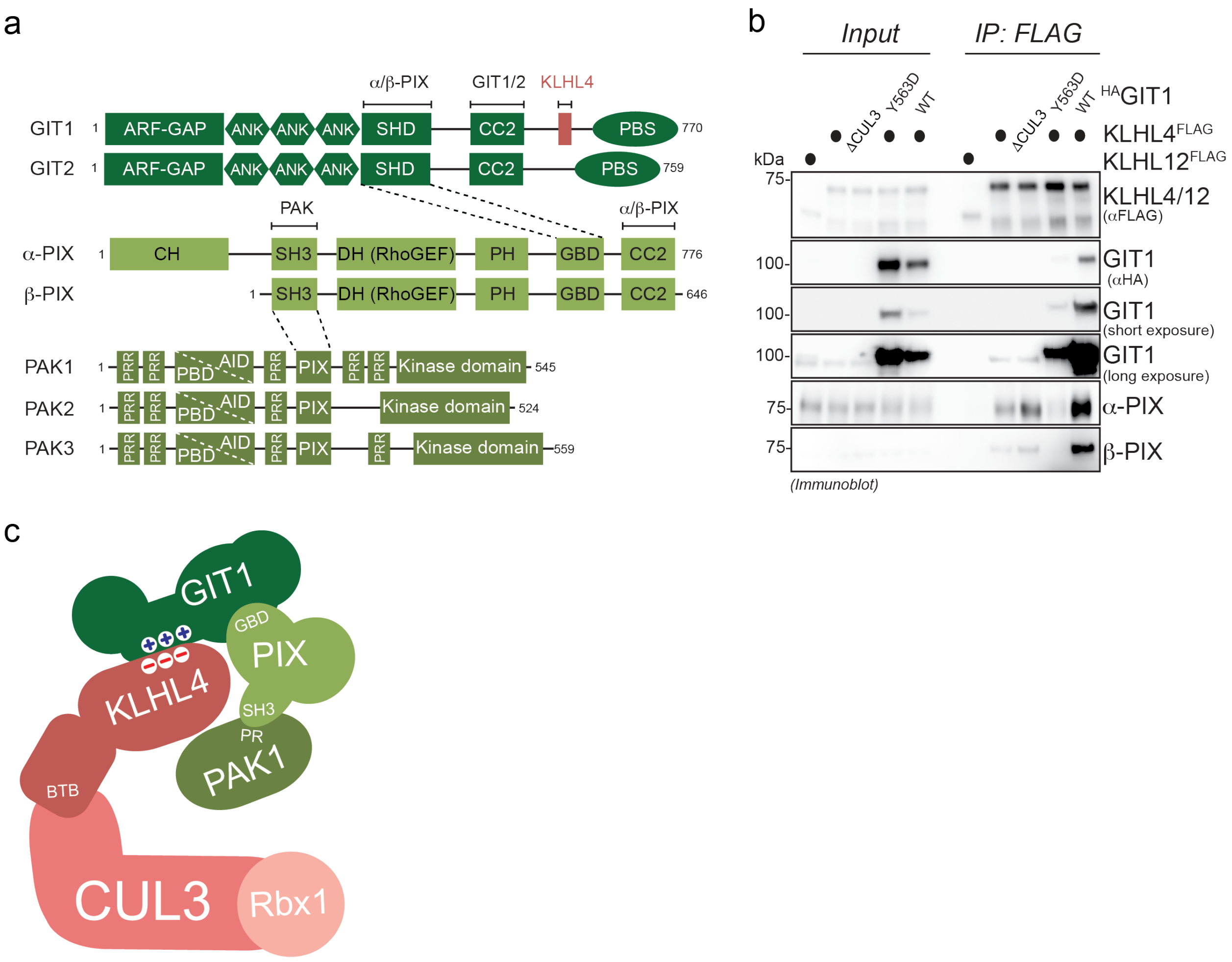

Supplemental Fig. 7: CRL3-KLHL4 utilizes GIT1-PIX complexes as substrate co-adaptors to recruit PAK1. a) Cartoon depicting domain structures of GIT, PIX, and group I PAK proteins. Highlighted are the known interaction domains through which GIT-PIX-PAK complexes can form. GIT proteins bind to the GIT-binding domain (GBD) of PIX proteins. GIT-PIX complexes can further associate to a proline-rich motif in group I PAKs (denoted as PIX) via SH3 domains of PIX proteins. KLHL4 can associate to these assemblies via a binding site in GIT1 (colored in red). **b**) GIT1 recruits PIX proteins to KLHL4. HEK293T cells were co-transfected with indicated FLAG-tagged KLHL4 or HA-tagged GIT1 constructs, lysed, and subjected to anti-FLAG IP followed by immunoblotting with indicated antibodies. KLHL12^{FLAG} served as specificity control for interactions. Compared to KLHL4^{FLAG} alone, co-expression of ^{HA}GIT1^{WT} increases binding of PIX proteins, while co-expression of the KLHL4-binding deficient ^{HA}GIT1^{Y563D} decreases binding of PIX proteins, indicating that they are sequestered. Immunoblots are representative of 2 biological replicates. **c**) Cartoon depicting a simplified model on how CRL3-KLHL4 uses GIT-PIX complexes as co-adaptors to bind PAK1.

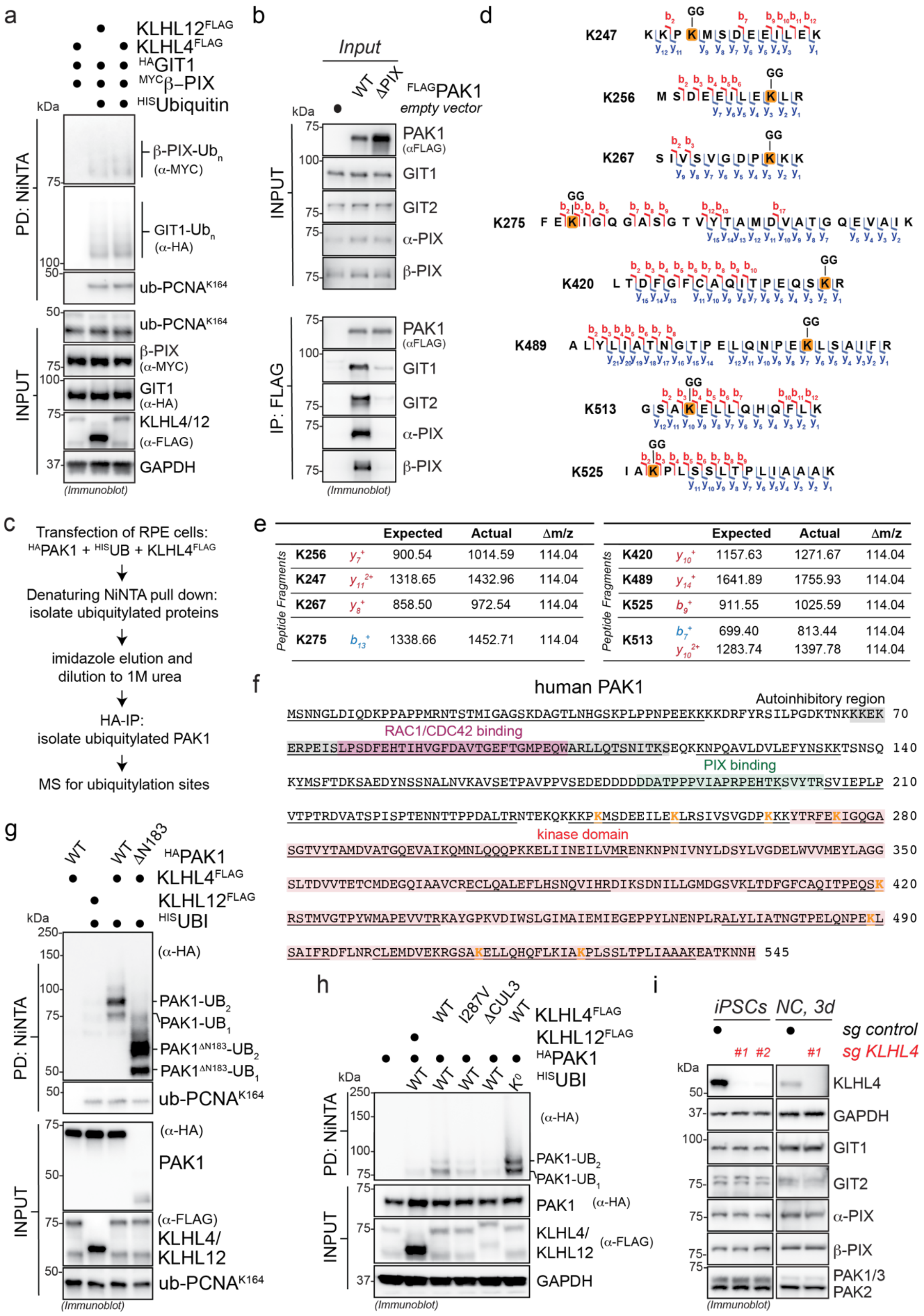

Supplemental Fig. 8: CRL3-KLHL4 utilizes GIT1-PIX complexes to recruit and multi-monoubiquitylate PAK1 in its C-terminus. a) GIT1/β-PIX complexes do not

undergo CRL3-KLHL4-dependent ubiquitylation in cells. Immunoblot analysis of denaturing ^{HIS}ubiquitin pull downs from RPE-1 cells expressing denoted combinations of constructs using the indicated antibodies. PCNA = control for general ubiquitylation efficiency. n = 1 replicate. **b**) Verification that a previously published PIX-binding-deficient PAK1 mutant cannot engage GIT-PIX complexes in cells. RPE-1 cells were transfected with ^{FLAG}PAK1^{WT} or ^{FLAG}PAK1$^{PRP192\text{-}194AAA}$ (ΔPIX), lysed, and subjected to anti-FLAG IP followed by immunoblotting with indicated antibodies. **c**) Outline of the experimental approach to map ubiquitylation sites in PAK1. **d**) Overview of the 8 ubiquitylated PAK1 peptides identified by the approach described in panel c. Ubiquitylated lysine residues are colored in orange and labeled with a diGly remnant (GG). Detected b and y ions for each peptide are highlighted in red and dark blue, respectively. **e**) Table summarizing the masses of fragments of each ubiquitylated PAK1 peptide carrying the diGly remnant, as revealed by the Δm/z of 114.04Da. **f**) Schematic overview of human PAK1, highlighting the sequence being covered by our MS analysis (overall coverage is ~70%) and indicating functional motifs and domains and the mapped ubiquitylation sites (orange), which cluster within or just outside of the C-terminal kinase domain. **g**) N-terminally truncated PAK1 (ΔN183) still undergoes CRL3-KLHL4-mediated ubiquitylation, indicating ubiquitylation at the C-terminus. Ubiquitylated proteins were purified under denaturing conditions from RPE-1 cells expressing ^{HIS}ubiquitin and denoted combinations of ^{HA}PAK1, ^{HA}PAK1ΔN183, KLHL12^{FLAG}, and KLHL4^{FLAG}. Modified proteins were detected by immunoblotting using the indicated antibodies. n = 1 replicate. **h**) CRL3-KLHL4 multi-monoubiquitylates PAK1 in cells. Ubiquitylated proteins were purified under denaturing conditions from RPE-1 cells expressing ^{HIS}Ubiquitin WT or chain formation-deficient ^{HIS}Ubiquitin (K^0) and denoted combinations of ^{HA}PAK1, KLHL12^{FLAG}, and KLHL4^{FLAG} variants. Modified proteins were detected by immunoblotting using the indicated antibodies. WT and chain formation-deficient ^{HIS}Ubiquitin support the same pattern of PAK1 modification, suggesting that these are multi-monoubiquitylation events. n = 1 replicate**. i**) KLHL4 depletion does not change steady state levels of components of the GIT-PIX-PAK signaling module. Control or KLHL4-depleted CRISPRi iPSCs or cells undergoing neural conversion for 3d were lysed and subjected to immunoblotting using the indicated antibodies. n =2 biological replicates.

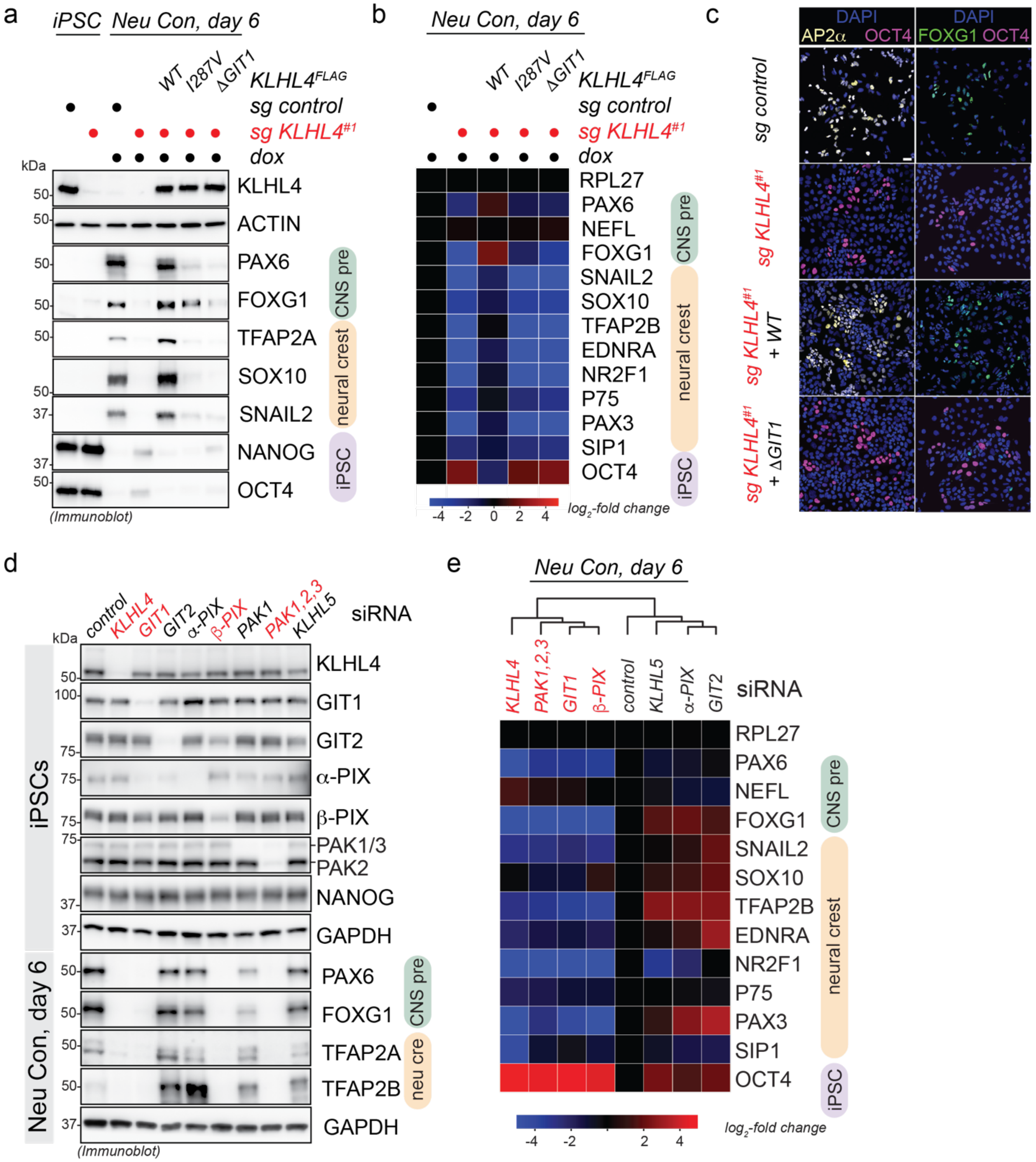

Supplemental Fig. 9: CRL3-KLHL4 controls ectodermal differentiation through the GIT-PIX-PAK signaling module. a) The GIT1-binding deficient mutant of KLHL4 is not able to support neural conversion. Control or KLHL4-depleted CRISPRi iPSCs stably expressing sgRNA-resistant and doxycycline-inducible wildtype KLHL4^{FLAG} (WT), patient variant KLHL4^{FLAG} (I287V), or GIT-PIX-PAK-binding deficient KLHL4^{FLAG} (ΔGIT1) were treated with doxycycline (dox) and subjected to neural conversion for 6 days.

Differentiation was monitored by immunoblotting using indicated antibodies against lineage markers (iPSC = purple; CNS precursor = green; neural crest = orange; ACTIN = loading control). Immunoblots are representative of 2 biological replicates. **b**) Control or KLHL4-depleted CRISPRi iPSCs were reconstituted and differentiated as described in panel A, followed by qRT-PCR analysis for expression of indicated lineage markers. Marker expression was normalized to control iPSCs. (RPL27 = endogenous control, n=2 biological replicates with 3 technical replicates each). **c**) Control or KLHL4-depleted CRISPRi iPSCs were reconstituted as described in panel A, treated with dox, and subjected to neural conversion for 8d followed by immunofluorescence microscopy using indicated antibodies. Representative images of n= 4 biological replicates are shown. Scale Bar = 40µm. **d**) Depletion of GIT1, β-PIX, and group I PAKs (PAK1,2,3) phenocopies the aberrant neural conversion program observed upon KLHL4 reduction. Control CRISPR iPSCs were depleted of endogenous KLHL4 or indicated proteins using siRNA, subjected to neural conversion for 6d, and analyzed by immunoblotting. Conditions that phenocopy KLHL4 depletion are highlighted in red. Immunoblots are representative of 1 biological replicate. **e**) Control CRISPR iPSCs were depleted of endogenous KLHL4 or indicated proteins using siRNA and cells were subjected to neural conversion for 6d, followed by qRT-PCR analysis for expression of CNS precursor markers (green), neural crest markers (orange), and iPSC markers (purple). Marker expression was normalized to control iPSCs and RPL27 was used as endogenous control. Heatmap depicts the average of 2 biological replicates with 3 technical replicates each.

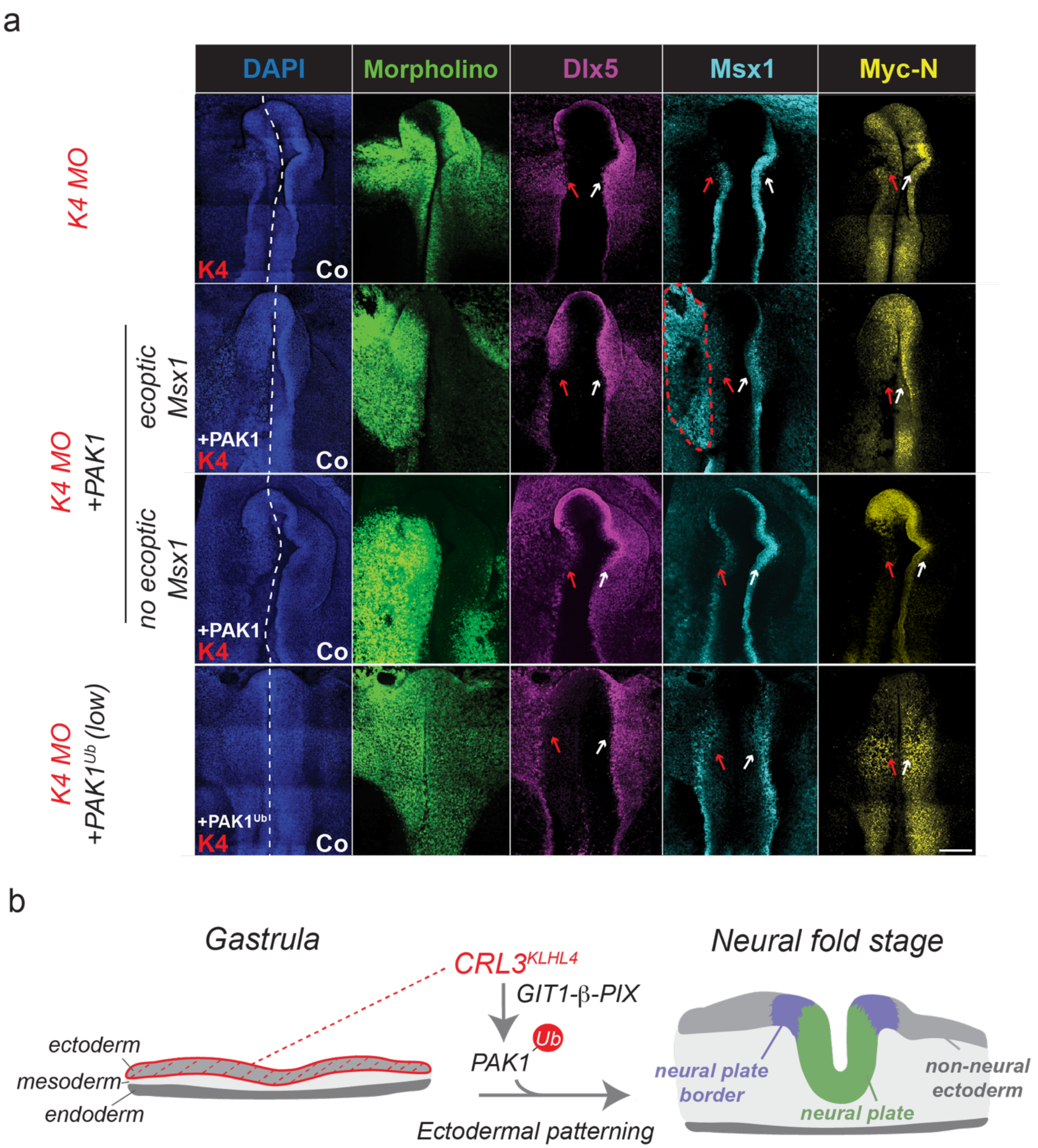

Supplemental Fig. 10: CRL3-KLHL4 controls ectodermal development through monoubiquitylation of PAK1. a) Co-expression of ubiquitylated PAK1 rescues the loss of KLHL4 phenotype as shown by expression of *Dlx5*, *Msx1* and *Myc-N*, the respective markers for the non-neural ectoderm, neural plate border/neural crest and neural plate as indicated by the arrows in whole mount embryos after HCR FISH. Morpholino-induced loss of KLHL4 phenotype is not rescued by the non-ubiquitylated WT PAK, as shown by decreased expression levels of the respective markers comparable to the

phenotype caused by KLHL4 MO alone. Additionally, expression of PAK1 occasionally induces ectopic expression of Msx1 next to the neural plate border region (circled area) as highlighted by two different outcomes of the PAK1 rescue. Representative images of n=6 control, 5 KLHL4 MO, 4 KLHL4 MO + PAK1, 7 KLHL4 MO + PAK1-Ub embryos are shown. Scale Bar = 250μm **b**) Model illustrating how CRL3-KLHL4, expressed specifically in the anterior ectoderm of the developing embryo, utilizes GIT1-β-PIX complexes as co-adaptors to ubiquitylate PAK1 to coordinate ectodermal domain specification and neurulation.

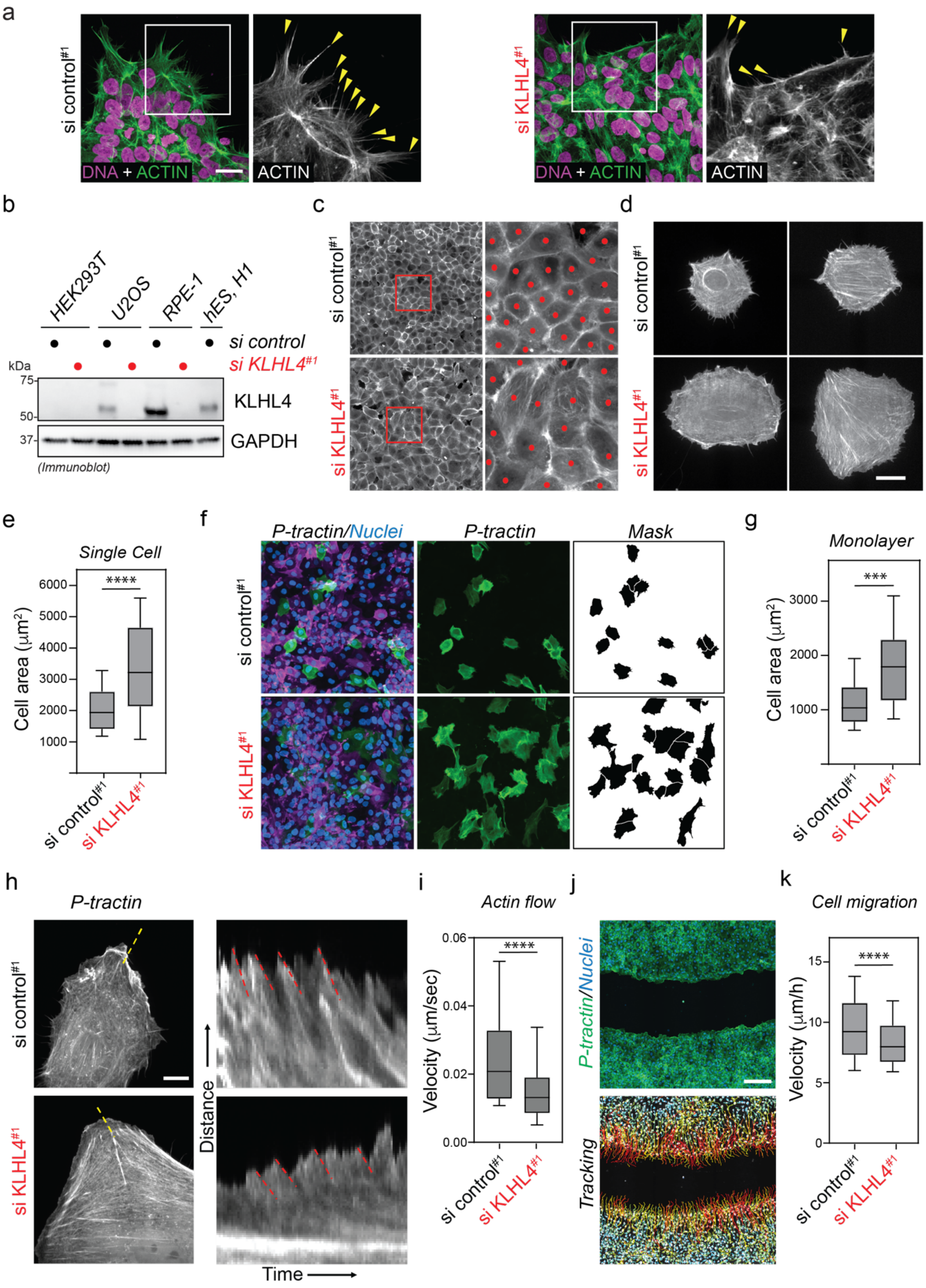

Supplemental Fig. 11: KLHL4 controls epithelial cell morphology and actin flow rates. a) KLHL4 regulates colony morphology and actin protrusions (arrowheads in

inset), as evidence by immunofluorescence microscopy of control and KLHL4-depleted hESCs (H1 line). Representative images of n=3 biological replicates are shown. Scale Bar = 20µm. **b**) RPE-1 cells express high levels of KLHL4. Anti-KLHL4 immunoblot analysis of cell lysates transfected with control or KLHL4 siRNA for 72h. GAPDH = loading control. n = 1 replicate. **c**) Representative examples of siControl$^{\#1}$ (top) and siKLHL4$^{\#1}$ (bottom) RPE-1 cells expressing EGFP-P-tractin, demonstrating the change in cell area and concomitant cell packing common in cell monolayers. n=4 biological replicates. **d**) Representative examples of siControl$^{\#1}$ (top) and siKLHL4$^{\#1}$ (bottom) RPE-1 cells expressing EGFP-P-tractin, demonstrate the change in cell area is also observed in single cell plating experiments. n= 4 biological replicates**. e**) Quantification of single cell area between siControl$^{\#1}$- and siKLHL4$^{\#1}$-treated RPE-1 cells. n=4, at least 36 cells were quantified per condition. p<0.0001, unpaired t-test. center line=median, whiskers=5-95 percentile **f**) Examples of siControl$^{\#1}$ (top) and siKLHL4$^{\#1}$ (bottom) RPE-1 cells plated as a monolayer expressing either EGFP (green) or mScarlet (magenta)-P-tractin. Nuclei (Spy 650 DNA) are shown in blue. EGFP-P-tractin cells (middle) were thresholded, masked and segmented (right) to demonstrate the enlarged cell area. **g**) Quantification of monolayer cell area between siControl$^{\#1}$ and siKLHL4$^{\#1}$ treated RPE-1 cells. n=4, at least 280 cells were quantified per condition. $p < 0.0001$. unpaired t-test. center line=median, whiskers=10-90 percentile **h**) siControl$^{\#1}$ (top) and siKLHL4$^{\#1}$ (bottom) RPE-1 cells expressing EGFP-P-tractin were imaged at high resolution rapidly over time to observe the dynamic flow of extending lamellipodia. Dashed yellow lines mark the position of each kymograph (right). Dashed red lines indicate the local flow rate of actin during protrusion/retraction cycles of the lamellipodia. **I**) Quantification of actin flow rate between sicontrol$^{\#1}$- and siKLHL4$^{\#1}$-treated RPE-1 cells. n=10 cells per condition, at least 59 kymographs were quantified per condition. p=0.0002, unpaired t-test. center line=median, whiskers=10-90 percentile. **j**) Example image on a monolayer wound assay showing siKLHL4$^{\#1}$ treated RPE-1 cells (top) expressing EGFP-P-tractin (green) with marked nuclei (Spy650 DNA, blue). Bottom images shows the nuclear tracking used to quantify cell migration rate. **k**). Quantification of cell migration rates between siControl$^{\#1}$- and siKLHL4$^{\#1}$-treated RPE-1 cells. n=4, at least 10000 cells were quantified per condition. $p<0.0001$, unpaired t-test. center line=median, whiskers=10-90 percentile. Scale bars: B; 20 µm, D; 100 µm, F; 10 µm, I: 200 µm.

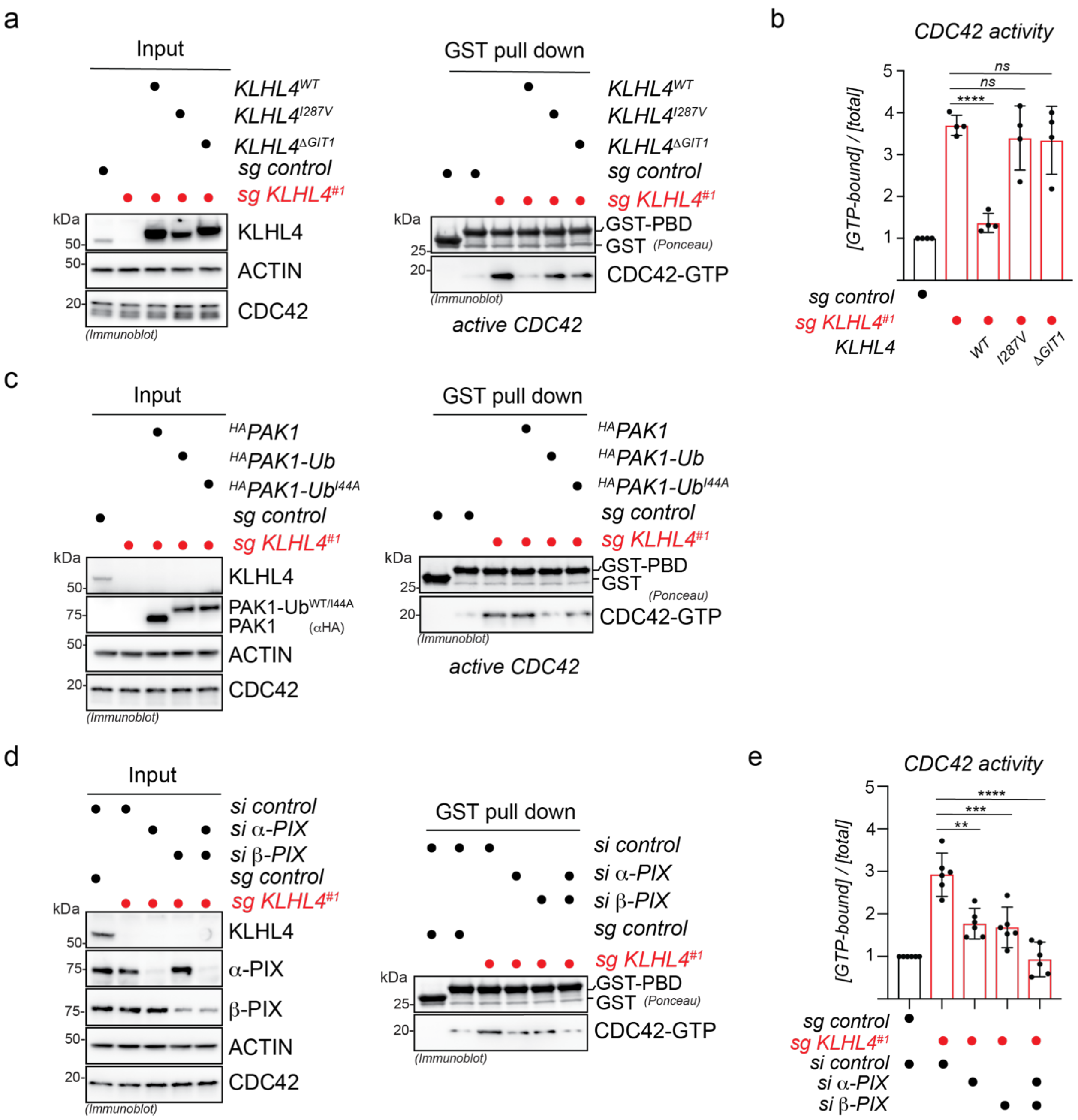

Supplemental Fig. 12: CRL3-KLHL4 mediated PAK1 ubiquitylation restricts CDC42 activity during ectodermal differentiation *in vitro*. a) CRL3-KLHL4 ubiquitylation activity and binding to the GIT-PIX-PAK module are required to restrict CDC42 activity. Control or KLHL4-depleted CRISPRi iPSCs were reconstituted with dox-inducible wildtype KLHL4^{FLAG} (WT), CUL3-binding deficient, patient variant KLHL4^{FLAG} (I287V), or GIT-PIX-PAK-binding deficient KLHL4^{FLAG} (ΔGIT1), treated with dox, and subjected to neural conversion for 3d. GTP-bound CDC42 was affinity-purified using GST-PBD followed by immunoblotting with indicated antibodies. GST was used as specificity control for binding. **b**) Quantification of the relative CDC42 activity of the

experiment in panel b. mean of n= 2 biological with 2 technical replicates each, error bars denote s.d., WT: p = 0.0001, I287V: p=0.9189, AP1: p=0.8648, one-way ANOVA. **c**) Ubiquitylated PAK1 can restore normal levels of CDC42 activity in KLHL4-deficient cells undergoing early stages of neural conversion. Control or KLHL4-depleted CRISPRi iPSCs were reconstituted with dox-inducible ^{HA}PAK1, ^{HA}PAK1-Ub, or ^{HA}PAK1-Ub^{I44A}, treated with dox and subjected to neural conversion for 3d. GTP-bound CDC42 was affinity-purified using GST-PBD followed by immunoblotting with indicated antibodies. GST was used as specificity control for binding. n = 3 biological replicates that are quantified in Figure 5c. **d**) Co-depletion of α– and β–PIX can restore normal levels of CDC42 activity in KLHL4-deficient cells undergoing early stages of neural conversion. Control or KLHL4-depleted CRISPRi iPSCs were depleted of endogenous α- and/or β-PIX using siRNAs and subjected to neural conversion for 3d. GTP-bound CDC42 was affinity-purified using GST-PBD followed by immunoblotting with indicated antibodies. GST was used as specificity control for binding. **e**) Quantification of the relative CDC42 activity of the experiment in panel e. mean of n= 2 biological with 2 or 3 technical replicates each, error bars denote s.d., si α-PIX: p = 0.0015, si β-PIX: p = 0.007, si α/β-PIX: p < 0.0001, one-way ANOVA.

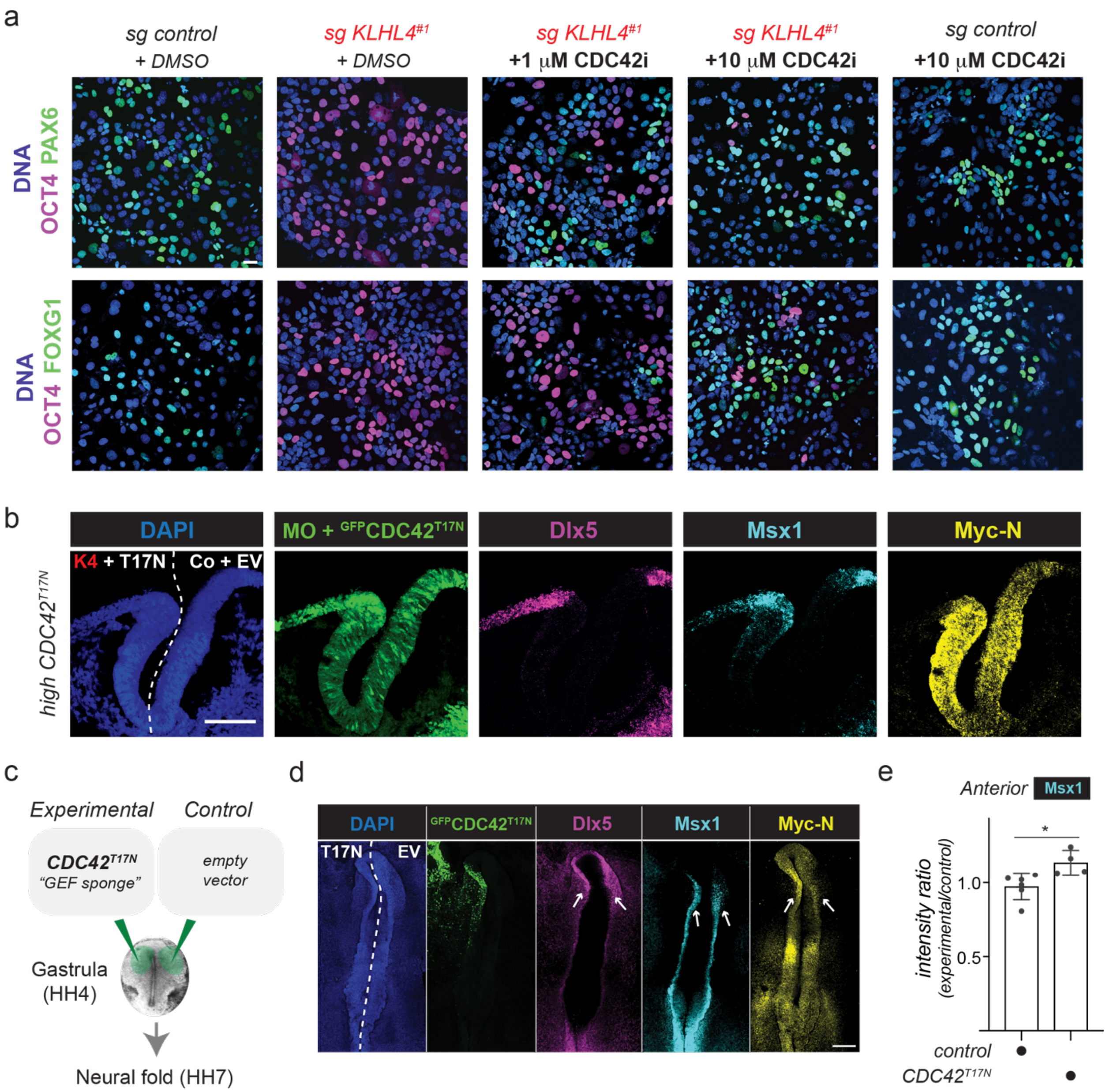

Supplemental Fig. 13: KLHL4-depletion induced ectodermal differentiation defects can be rescued by CDC42 inhibition. a) CDC42 inhibition rescues neural conversion of KLHL4-deficient iPSCs. Control or KLHL4-depleted CRISPRi iPSCs were treated with DMSO or indicated concentrations of the CDC42 inhibitor ML141 (CDC42i), subjected to neural conversion for 8d, and analyzed by immunofluorescence analysis for expression of the CNS precursor markers PAX6 and FOXG1 and the pluripotency marker OCT4. Representative images of n=2 biological replicates are shown. Scale bar = 10 μm. **b**) Cross-sections from the anterior midbrain region show that co-injection of KLHL4 MO with a high concentration of $CDC42^{T17N}$ results in premature, accelerated

folding of the neural tube on the experimental side, an opposite phenotype of the one caused by loss of KLHL4 (see Supplementary Fig 5e). The dotted line marks the midline. Representative images of n=4 biological replicates are shown. Scale Bar = 100 µm. **c**) Experimental design for panels D and E. ^{GFP}CDC42^{T17N} was electroporated into the epiblast in one side of the embryo (experimental) and an empty vector (EV) on the other side (control), and grown until they reach the neural fold stage (HH7). **d**) Expression of downstream signaling-deficient CDC42^{T17N} leads to aberrant ectodermal patterning during chick development. Embryos analyzed by HCR fluorescent *in situ* hybridization using probes for the neural plate border marker *Msx1*, the neural plate marker *Myc-N*, and the non-neural ectoderm marker *Dlx5*. Scale Bar = 250µm. Arrows point to the anterior neural folds that show premature folding on the experimental side. **e**) Graph depicts ratios of anterior *Msx1* fluorescence intensity of the experimental side relative to the control side of the embryo. n = 4 experimental ^{GFP}CDC42^{T17N} /EV and 6 control embryos (EV/EV), error bars = s.e.m., $p < 0.0219$, unpaired t-test.

Supplementary Note 1: Human disease variant screening identifies CRL3-KLHL4 as candidate regulator of ectodermal differentiation

A subset of neurodevelopmental and craniofacial disease-causing and disease-associated CUL3 variants are missense mutations and located in the domain of CUL3 that mediates interaction with BTB substrate adaptors (Fig. 1a, Supplementary Data 1). We hypothesized that these mutations could interfere with substrate adaptor binding and that we could use them to identify CRL3 complexes with previously unrecognized functions during ectodermal patterning. We thus immunoprecipitated FLAG-tagged wildtype (WT) CUL3 and variants associated with different types of developmental disease from human embryonic stem cells (hESCs) undergoing early stages of ectodermal differentiation and compared their interaction networks using mass spectrometry (MS) analysis (Supplementary Fig. 1a, Data 2). In general, while the CUL3 variants had only minor effects on interactions with CRL regulators such as CAND1 and CSN, they had a marked impact on BTB substrate adaptor binding compared to WT CUL3. In particular, for all tested CUL3 variants, interactions with some, but not all BTB proteins were altered, suggesting that dysregulation of particular CRL3 complexes might underlie their pathogenic effects on neural and neural crest development. In most cases, BTB interactions were reduced; however, two variants (CUL3 p.S53N and p.Y62F) also exhibited increased binding to a subset of BTB proteins, raising the possibility that for these mutations some of the disease phenotypes might originate from dominant negative mechanisms. Importantly, while the differentially bound substrate adaptor repertoire varied amongst different CUL3 mutants, we identified 15 BTB proteins that were reproducibly reduced more than 1.8-fold in at least 3 different CUL3 variants (Fig. 1b, Supplementary Fig. 1a,b), suggesting that loss of ubiquitylation activity of these particular CRL3 complexes might be a more common mechanism of how CUL3 mutations cause developmental diseases. Amongst these hits were some known regulators of ectodermal differentiation (including KCTD13[1], KLHL15[2], and ENC1[3]), but also several BTB proteins previously not linked to neuro- or craniofacial development. To prioritize amongst those novel candidate regulators of ectodermal differentiation, we reasoned that mutations in the CUL3 binding interface of the BTB protein itself might also lead to developmental disease. Therefore, we systematically searched exome

sequencing data of patients with undiagnosed developmental diseases for variants in BTB proteins focusing on missense mutations located in BTB and BACK domains (Fig 1a). We queried databases including DECIPHER[4], denovo-db[5] and utilized genematcher[6] to identify 18 such candidate disease-causing variants in 16 BTB proteins (Supplementary Data 3). While for most of these mutations we could not observe any obvious effects on BTB dimerization or CUL3 binding when analyzing FLAG-immunoprecipitation (IP) fractions from HEK 293T cells by immunoblotting (Supplementary Fig. 2a-h), we found that four variants (KLHL1 p.L305P, KLHL4 p.I287V, KLHL8 p.C87F, and KLHL36 p.T288M,) exhibited a notable reduction in interaction with CUL3 compared to their WT counterparts (Supplementary Fig. 2i-m). Strikingly, two of these variants were in *KLHL4* and *KLHL36*, genes that are restricted in heterozygous or hemi/homozygous loss-of-function mutations (*KLHL4* pLI = 0.66, o/e = 0.19; *KLHL36* pLI = 0.81, o/e=0.16*)* in healthy individuals and that encode for two poorly characterized BTB proteins that were also reduced in IPs of at least three developmental disease-associated CUL3 variants (Fig. 1b, Supplementary Fig. 2a,b, Data 2). This suggests that ubiquitylation activity of both, CRL3-KLHL4 and CRL3-KLHL36, are likely important for ectodermal development.

Supplementary Note 2: Detailed patient description

Subject 1 is a male child born of non-consanguineous caucasian parents. Pregnancy and delivery were uncomplicated and was born at 40+2 weeks with a birth weight of 2665 grams (7%,, SD, -1.5), height 49 cm (32%,, SD -.47), and head circumference 35 cm (66%, SD .42). Apgar scores after 5 and 10 minutes were 7 and 9. He had distinctive features with a relative large skull with wide sutures and fontanels, a small face with pointed nose, hypertelorism, micrognathia, low-set ears, hypermobile joints, irregular inplant of his toes, a single umbilical artery, and a small penis. He also has a perimembraneous ventricular septal defect. He has partial ocular albinism (no retinal pigment, little pigment in the iris) and bilateral congenital glaucoma, resulting in blindness besides some light perception. His psychomotor development was severely delayed. He has no speech and understands some phrases.

He had failure-to-thrive, resulting in a severe short stature with dystrophic build. He had very frequent ENT and pulmonary infections and has hearing loss after recurrent ear infections. At the age of 9 years, he had meningococcal meningitis. He was diagnosed with a common variable immunodeficiency. At the age of 9 years, head circumference was 52.3 cm (-0.4 SD). At 19 years, his height was 137 cm (-6.5 SD), his weight 28 kg (BMI 14.9 (-4,4 SD)).

CT cerebrum showed hypoplasia of the cerebellum and vermis, a megacysterna magna, a cavum septum pellucidum, and cerebral migration defects. He developed immune thrombopenic purpura (ITP) at the age of 17 years, followed by severe autoimmune hemolytic anemia six years later.

Karyotype was 46,XY, SNP array analysis didn't show CNVs. Sanger sequencing of *CASP10, FAS*, and *FASLG* for CVID/ALPS. Trio-WES revealed a maternal missense variant in *KLHL4* (NM_019117.4(KLHL4):c.859A>G p.(Ile287Val) ChrX(GRCh37):g.86873066A>G) and a homozygous variant in *VPS35L* (c.2627C>T: p.P876L).

He has one healthy brother. His other brother was affected and had a similar phenotype as he has, with a severe psychomotor retardation, a large VSD, recurrent (mainly pulmonary and skin) infections, a relative large skull with wide sutures and a small face with low-set ears, some hypertelorism, and retrognathia. He was dysmature at birth, with an average height and head circumference. His head circumference increased rapidly due to a severe communicating hydrocephalus. He had a growth retardation. Ophthalmologic examination was normal. At the age of 21 months, he died of a sepsis after cardiac operation. CT cerebrum showed some cerebellar hypoplasia. He had a normal male karyotype and no additional genetic testing was performed or possible to date.

Supplementary Note 3: Identification of components of the GIT-PIX-PAK signaling module as interactors and candidate substrates of CRL3-KLHL4

To identify binding partners and candidate substrates of KLHL4, we immunoprecipitated FLAG-tagged KLHL4 from HEK 293T cells and determined high confidence interaction partners (HCIPs) by mass spectrometry and compPASS analysis using top 5% of

interactors with highest Z-score and WD-score as thresholds[7,8] (Fig. 3a, Supplementary Data 4). For this, three independent KLHL4 immunoprecitations (IPs) were compared as replicates against a database of ~30 reference IPs. As expected, this revealed strong interactions of KLHL4 with CUL3 and other CRL3 components and regulators. In addition, while there were some interactors associated with the secretory pathway, we found that many KLHL4 HCIPs were related to the actin cytoskeleton. These included GIT1, GIT2, α-PIX, β-PIX and PAK1, components of the GIT-PIX-PAK signaling axis, which regulates various aspects of ectodermal differentiation by modulating the activity of the small GTPases CDC42 and RAC1 [9], and genetic lesions in this pathway are linked to diverse neurodevelopmental and craniofacial disorders [10-16] (Fig. 3a, Supplementary Data 5). To test whether components of this signaling module could be candidate substrates of CRL3-KLHL4, we reasoned that interactions should occur through KLHL4's substrate-binding domain and should not be affected by KLHL4 mutations that interfere with CUL3 binding. We thus repeated the mass spectrometry analysis with the KLHL4 p.I287V patient variant or a more severe CUL3-binding mutant (KLHL4ΔCUL3) and compared relative total spectral counts of HCIPs to WT KLHL4 (Fig. 3b). Indeed, while the majority of KLHL4 interactors were reduced in one or both CUL3-binding mutants, interaction with the GIT-PIX-PAK signaling module remained stable.

Supplementary references

1 Golzio, C. *et al.* KCTD13 is a major driver of mirrored neuroanatomical phenotypes of the 16p11.2 copy number variant. *Nature* **485**, 363-367, doi:10.1038/nature11091 (2012).

2 Hu, H. *et al.* X-exome sequencing of 405 unresolved families identifies seven novel intellectual disability genes. *Mol Psychiatry* **21**, 133-148, doi:10.1038/mp.2014.193 (2016).

3 Hernandez, M. C. *et al.* ENC-1: a novel mammalian kelch-related gene specifically expressed in the nervous system encodes an actin-binding protein. *J Neurosci* **17**, 3038-3051 (1997).

4 Firth, H. V. *et al.* DECIPHER: Database of Chromosomal Imbalance and Phenotype in Humans Using Ensembl Resources. *Am J Hum Genet* **84**, 524-533, doi:10.1016/j.ajhg.2009.03.010 (2009).

5 Turner, T. N. *et al.* denovo-db: a compendium of human de novo variants. *Nucleic Acids Res* **45**, D804-D811, doi:10.1093/nar/gkw865 (2017).

6 Sobreira, N., Schiettecatte, F., Valle, D. & Hamosh, A. GeneMatcher: a matching tool for connecting investigators with an interest in the same gene. *Hum Mutat* **36**, 928-930, doi:10.1002/humu.22844 (2015).
7 Huttlin, E. L. *et al.* The BioPlex Network: A Systematic Exploration of the Human Interactome. *Cell* **162**, 425-440, doi:10.1016/j.cell.2015.06.043 (2015).
8 Sowa, M. E., Bennett, E. J., Gygi, S. P. & Harper, J. W. Defining the human deubiquitinating enzyme interaction landscape. *Cell* **138**, 389-403, doi:10.1016/j.cell.2009.04.042 (2009).
9 Zhou, W., Li, X. & Premont, R. T. Expanding functions of GIT Arf GTPase-activating proteins, PIX Rho guanine nucleotide exchange factors and GIT-PIX complexes. *J Cell Sci* **129**, 1963-1974, doi:10.1242/jcs.179465 (2016).
10 Kim, M. J. *et al.* Functional analysis of rare variants found in schizophrenia implicates a critical role for GIT1-PAK3 signaling in neuroplasticity. *Mol Psychiatry* **22**, 417-429, doi:10.1038/mp.2016.98 (2017).
11 Kutsche, K. *et al.* Mutations in ARHGEF6, encoding a guanine nucleotide exchange factor for Rho GTPases, in patients with X-linked mental retardation. *Nat Genet* **26**, 247-250, doi:10.1038/80002 (2000).
12 Harms, F. L. *et al.* Activating Mutations in PAK1, Encoding p21-Activated Kinase 1, Cause a Neurodevelopmental Disorder. *Am J Hum Genet* **103**, 579-591, doi:10.1016/j.ajhg.2018.09.005 (2018).
13 Allen, K. M. *et al.* PAK3 mutation in nonsyndromic X-linked mental retardation. *Nat Genet* **20**, 25-30, doi:10.1038/1675 (1998).
14 Wang, Y. *et al.* PAK2 Haploinsufficiency Results in Synaptic Cytoskeleton Impairment and Autism-Related Behavior. *Cell Rep* **24**, 2029-2041, doi:10.1016/j.celrep.2018.07.061 (2018).
15 Reijnders, M. R. F. *et al.* RAC1 Missense Mutations in Developmental Disorders with Diverse Phenotypes. *Am J Hum Genet* **101**, 466-477, doi:10.1016/j.ajhg.2017.08.007 (2017).
16 Martinelli, S. *et al.* Functional Dysregulation of CDC42 Causes Diverse Developmental Phenotypes. *Am J Hum Genet* **102**, 309-320, doi:10.1016/j.ajhg.2017.12.015 (2018).
