# Supplementary material for: A ubiquitin-based effector-to-inhibitor switch coordinates early brain, craniofacial, and skin development
Source: Nat Commun. 2023 Jul 26;14:4499. doi: 10.1038/s41467-023-40223-y (PMC10371987; doi:10.1038/s41467-023-40223-y)
Supplement: Supplementary file 8 — Reporting Summary [file 41467_2023_40223_MOESM8_ESM.pdf]

nature portfolio

Corresponding author(s): Achim Werner and Laura Kerosuo (NCOMMS-22-44020A)

Last updated by author(s): 7/12/2023

Reporting Summary

Nature Portfolio wishes to improve the reproducibility of the work that we publish. This form provides structure for consistency and transparency in reporting. For further information on Nature Portfolio policies, see our Editorial Policies and the Editorial Policy Checklist.

Statistics

For all statistical analyses, confirm that the following items are present in the figure legend, table legend, main text, or Methods section.

n/a	Confirmed	
☐	☒	The exact sample size (*n*) for each experimental group/condition, given as a discrete number and unit of measurement
☐	☒	A statement on whether measurements were taken from distinct samples or whether the same sample was measured repeatedly
☐	☒	The statistical test(s) used AND whether they are one- or two-sided<br>*Only common tests should be described solely by name; describe more complex techniques in the Methods section.*
☒	☐	A description of all covariates tested
☒	☐	A description of any assumptions or corrections, such as tests of normality and adjustment for multiple comparisons
☐	☒	A full description of the statistical parameters including central tendency (e.g. means) or other basic estimates (e.g. regression coefficient) AND variation (e.g. standard deviation) or associated estimates of uncertainty (e.g. confidence intervals)
☐	☒	For null hypothesis testing, the test statistic (e.g. *F*, *t*, *r*) with confidence intervals, effect sizes, degrees of freedom and *P* value noted<br>*Give P values as exact values whenever suitable.*
☒	☐	For Bayesian analysis, information on the choice of priors and Markov chain Monte Carlo settings
☒	☐	For hierarchical and complex designs, identification of the appropriate level for tests and full reporting of outcomes
☒	☐	Estimates of effect sizes (e.g. Cohen's *d*, Pearson's *r*), indicating how they were calculated

Our web collection on statistics for biologists contains articles on many of the points above.

Software and code

Policy information about availability of computer code

Data collection	Protein identification was carried out using Proteome Discoverer (V2.4) software (Thermo Scientific).
Data analysis	Mass spec data analysis: CompPASS (Sowa et al, Cell, 2009, PMID: 19615732), Scaffold4; Image analysis: Fiji/ImageJ 2.9.0; Statistical analysis: GraphPad Prism 9

For manuscripts utilizing custom algorithms or software that are central to the research but not yet described in published literature, software must be made available to editors and reviewers. We strongly encourage code deposition in a community repository (e.g. GitHub). See the Nature Portfolio guidelines for submitting code & software for further information.

Data

Policy information about availability of data

All manuscripts must include a data availability statement. This statement should provide the following information, where applicable:

- Accession codes, unique identifiers, or web links for publicly available datasets
- A description of any restrictions on data availability
- For clinical datasets or third party data, please ensure that the statement adheres to our policy

All data supporting the findings are available in the main text, the supplementary material file, and the source data file. For identifying candidate disease causing variants, we queried the public databases DECIPHER (https://www.deciphergenomics.org) and denovo-db (https://denovo-db.gs.washington.edu/denovo-db/) and

also utilized genematcher (https://genematcher.org). To visualize the position of disease-associated CUL3 variants in the CUL3-BTB interface, we used the crystal structure of the KLHL11-CUL3 complex with the pdb entry 4APF [https://doi.org/10.2210/pdb4APF/pdb]. Proteomics data are provided in Supplementary Data 2 and 4 and were deposited into the Mass Spectrometry Interactive Virtual Environment (MassIVE) under the accession number MSV000090711. Requests for resources and reagents should be directed to and will be fulfilled by the lead contact, Achim Werner (Achim.werner@nih.com).

Human research participants

Policy information about studies involving human research participants and Sex and Gender in Research.

Reporting on sex and gender	does not apply
Population characteristics	does not apply
Recruitment	does not apply
Ethics oversight	The patient carrying the KLHL4 variant was consented for clinical and research-based exome sequencing as well as for research-based phenotyping through the University Medical Centre Utrecht, The Netherlands. Research with patient samples complied with all relevant ethical regulations of the University Medical Centre Utrecht, The Netherlands. A consent form to allow for publication was signed by the parents and makes part of the medical record present at the University Medical Centre Utrecht, the Netherlands. The patient was male (as determined by karyotyping)

Note that full information on the approval of the study protocol must also be provided in the manuscript.

Field-specific reporting

Please select the one below that is the best fit for your research. If you are not sure, read the appropriate sections before making your selection.

☒ Life sciences ☐ Behavioural & social sciences ☐ Ecological, evolutionary & environmental sciences

For a reference copy of the document with all sections, see nature.com/documents/nr-reporting-summary-flat.pdf

Life sciences study design

All studies must disclose on these points even when the disclosure is negative.

Sample size	Measures taken to verify reproducibility included to perform experiments at least three times (for exceptions, see respective figure legends, in which the number of repetitions are specified for each experiment). No statistical analysis was performed to predetermine sample size, but three biological replicates is considered standard practice for most biochemical and cellular assays.
Data exclusions	No data was excluded from this study
Replication	All of the experiments in this manuscript were replicated at least once and whenever feasible experiments were repeated in biological triplicates and quantified as described in the figure legends
Randomization	The samples were not randomized in our study. The main task was to identify and characterize protein-protein interactions by biochemical and cell biological approaches, so it was not possible to rearrange sample orders randomly.
Blinding	Blinding was not used in our study. The main task was to identify and characterize protein-protein interactions by in vitro and cell-based approaches. In contrast to medical studies with human subjects in which placebo and nocebo effects are to be expected, these effects do not occur in our studies with isolated molecules.

Reporting for specific materials, systems and methods

We require information from authors about some types of materials, experimental systems and methods used in many studies. Here, indicate whether each material, system or method listed is relevant to your study. If you are not sure if a list item applies to your research, read the appropriate section before selecting a response.

Materials & experimental systems

n/a	Involved in the study
	☒ Antibodies
	☒ Eukaryotic cell lines
☒	Palaeontology and archaeology
☒	Animals and other organisms
☒	Clinical data
☒	Dual use research of concern

Methods

n/a	Involved in the study
☒	ChIP-seq
☒	Flow cytometry
☒	MRI-based neuroimaging

Antibodies

Antibodies used

All antibodies used in this study are summarized with their name, vendor, catalogue number, dilution used in specific application, and specific antibody registry identifier number in Extended data file 5.

Validation

We made monoclonal antibodies against KLHL4 in this study and validated their specificity by immunoblotting of control and KLHL4-depleted cells as shown in e.g. Figure 1e. All commercial antibodies were validated by the manufacturer in the respective application as detailed in the following:

Mouse monoclonal anti-beta-ACTIN (clone C4) MP Biomedicals, Cat#: 691001, RRID: AB_2335127: this antibody has been verified in immunoblotting, immunohistochemistry, immunofluorescence, and ELISA as stated by the supplier

Rabbit monoclonal anti-HA-Tag (C29F4) Cell Signaling, Cat#: 3724; RRID: AB_10693385: this antibody has been verified in immunoblotting, immunoprecipitation, immunohistochemistry, immunofluorescence, flowcytometry, and chromatin immunoprecipitation as stated by the supplier

Mouse anti-HA.11 antibody, BioLegend, Cat#: PRB-101P: this antibody has been verified in immunoblotting, immunoprecipitation as stated by the supplier

Rabbit monoclonal anti-Flag DYKDDDDK Tag, Cell Signaling, Cat#: 2368: this antibody has been verified in immunoblotting, immunoprecipitation, and immunofluorescence as stated by the supplier

Mouse monoclonal anti-Flag DYKDDDDK Tag, Sigma, Cat#: F3165:
this antibody has been verified in immunoblotting, immunocytochemistry, immunoprecipitation, ELISA, EIA, Chromatin immunoprecipitation, immunofluorescence electron microscopy, flow cytometry, and super shift assays as stated by the supplier

Mouse monoclonal anti-FLAG clone M2, Sigma-Aldrich, Cat#: F1804; RRID: AB_262044: this antibody has been verified in immunoblotting, immunoprecipitation , immunohistochemistry, immunofluorescence, and immunocytochemistry as stated by the supplier

Mouse monoclonal anti-MYC, Cell Signaling, Cat#:2040; RRID: AB_2148465: this antibody has been verified in immunoblotting as stated by the supplier

Rabbit polyclonal anti-CUL3, Bethyl Cat#: A301-109A; RRID: AB_873023: this antibody has been verified in immunoblotting, immunohistochemistry, immunoprecipitation as stated by the supplier

Rabbit monoclonal anti-GAPDH, Cell Signaling, Cat#:14C10; RRID: AB_561053:
this antibody has been verified in immunofluorescence and Flow-Cytometry as stated by the supplier

Rabbit monoclonal anti-CAND1, Cell Signaling, Cat#: 8759S (D1F2); RRID: AB_11178669: this antibody has been verified in immunoblotting, immunohistochemistry, immunoprecipitation as stated by the supplier

Mouse monoclonal anti-KLHL12, Cell Signaling, Cat#:9406S (2G2); RRID: AB_2797699: this antibody has been verified in immunoblotting as stated by the supplier

Rabbit monoclonal anti-ubiquityl-PCNA(Lys164), Cell Signaling, Cat#:13439, RRID: AB_2798219: this antibody has been verified in immunoblotting, immunoprecipitation as stated by the supplier

Mouse monoclonal anti-Ubiquitin (P4D1), Cell Signaling, Cat#: 3936; AB_331292:
this antibody has been verified in immunoblotting, immunohistochemistry as stated by the supplier

Rabbit polyclonal anti-KBTBD7, abcam, Cat#: ab230126: this antibody has been verified in immunoblotting, immunohistochemistry as stated by the supplier

Mouse monoclonal anti-KLHL4 generated with GenScript, this study: we have verified this antibody in immunoblotting and immunoprecipitation. It does not work in immunofluorescence

Rabbit monoclonal anti-PAX6, Cell Signaling, Cat#:60433, RRID: AB_2797599

this antibody has been verified in immunoblotting, immunohistochemistry, immunofluorescence, and immunoprecipitation as stated by the supplier

Rabbit polyclonal anti-FOXG1, Abcam, Cat#: ab18259; RRID: AB_732415: this antibody has been verified in immunoblotting, and immunohistochemistry as stated by the supplier

Mouse monoclonal anti-TFAP2A, Novus Cat#: NB100-74359; RRID: AB_1048155: this antibody has been verified in immunoblotting, immunohistochemistry, immunofluorescence as stated by the supplier

Rabbit polyclonal anti-TFAP2B, Cell Signaling, Cat#: 2509; RRID: AB_2058198: this antibody has been verified in immunoblotting, immunohistochemistry, and immunofluorescence as stated by the supplier

Rabbit monoclonal anti-SOX10, Cell Signaling, Cat#: 89356; RRID: AB_2792980: this antibody has been verified in immunoblotting, immunohistochemistry, immunofluorescence as stated by the supplier

Rabbit monoclonal anti-SLUG/SNAIL2, Cell Signaling, Cat#: 9585; RRID: AB_2239535: this antibody has been verified in immunoblotting, immunoprecipitation, flow cytometry, and immunofluorescence as stated by the supplier

Rabbit monoclonal anti-NANOG, Cell Signaling, Cat#: 4903; RRID: AB_10559205: this antibody has been verified in immunoblotting, immunohistochemistry, immunofluorescence as stated by the supplier

Goat polyclonal anti-OCT4 Santa Cruz Biotechnology, Inc., Cat#: sc-8628; RRID:AB_653551: this antibody has been verified in immunoblotting, immunohistochemistry, immunofluorescence, immunoprecipitation, ELISA and flow cytometry as stated by the supplier

Rabbit polyclonal anti-GIT1, Cell Signaling, Cat#:2919; RRID: AB_2109982: this antibody has been verified in immunoblotting, immunoprecipitation as stated by the supplier

Rabbit polyclonal anti-GIT2, Cell Signaling, Cat#:6953; RRID: AB_10828712: this antibody has been verified in immunoblotting as stated by the supplier

Rabbit monoclonal Cool2/alpha-PIX, Cell Signaling, Cat#:4573; RRID: AB_2060193: this antibody has been verified in immunoblotting, and immunofluorescence as stated by the supplier

Rabbit polyclonal Cool1/beta-PIX Cell Signaling, Cat#:4515; RRID: AB_2274365: this antibody has been verified in immunoblotting, immunoprecipitation as stated by the supplier

Mouse monoclonal anti-PAK1,2,3 Santa Cruz Biotechnology, Inc., Cat#:sc-166174; RRID:AB_2160693: this antibody has been verified in immunoblotting, immunohistochemistry, immunoprecipitation, ELISA, Flow cytometry, and immunofluorescence as stated by the supplier

Rabbit monoclonal anti-CDC42, Cell Signaling, Cat#:2466; RRID: AB_2078082: this antibody has been verified in immunoblotting as stated by the supplier

Rabbit monoclonal anti-RAC1/2/3 Cell Signaling, Cat#:2465; RRID: AB_2176152: this antibody has been verified in immunoblotting as stated by the supplier

Rabbit monoclonal anti-RhoA, Cell Signaling, Cat#:2117; RRID: AB_10693922: this antibody has been verified in immunoblotting as stated by the supplier

Mouse monoclonal anti-PAX7, DSHB, Cat# pax7, RRID: AB_528428: this antibody has been verified in immunoblotting, immunohistochemistry, immunofluorescence Chromatin immunoprecipitation, flow cytometry, immunoprecipitation, FFPE, and gel super shift as stated by the supplier

In addition, we independently verify the following antibodies in immunoblotting using shRNA mediated knock down in our manuscript: anti-GIT1, anti-GIT2, anti-alpha-PIX, anti-beta-PIX, and anti-PAK1/2/3 (Supplementary Figure 9d).

Eukaryotic cell lines

Policy information about cell lines and Sex and Gender in Research

Cell line source(s)	HEK293T (ATTC: CRL-3216) , hTERT RPE-1 cells (ATTC: CRL-4000) hES H1 cells ((WA01) WiCell), iPSC line dCas9-KRAB WTC (Coriell: AICS-0090-391)
Authentication	The cell lines were purchased from the indicated supplier, expanded, and not further authenticated
Mycoplasma contamination	All cell lines were tested negative for mycoplasma contamination
Commonly misidentified lines (See ICLAC register)	No commonly misidentified cell lines were used.
